# CD40×HER2 bispecific antibody overcomes the CCL2-induced trastuzumab resistance in HER2-positive gastric cancer

**DOI:** 10.1136/jitc-2022-005063

**Published:** 2022-07-15

**Authors:** Weilin Sun, Xi Wang, Daohan Wang, Li Lu, Hai Lin, Zhaoxiong Zhang, Yangpu Jia, Xinyang Nie, Tong Liu, Weihua Fu

**Affiliations:** Department of General Surgery, Tianjin Medical University General Hospital, Tianjin, China

**Keywords:** gastrointestinal neoplasms, immunotherapy, macrophages, tumor microenvironment

## Abstract

**Background:**

There was much hard work to study the trastuzumab resistance in HER2-positive gastric cancer (GC), but the information which would reveal this abstruse mechanism is little. In this study, we aimed to investigate the roles of tumor cell-derived CCL2 on trastuzumab resistance and overcome the resistance by treatment with the anti-CD40-scFv-linked anti-HER2 (CD40 ×HER2) bispecific antibody (bsAb).

**Methods:**

We measured the levels of CCL2 expression in HER2-positive GC tissues, and revealed biological functions of tumor cell-derived CCL2 on tumor-associated macrophages (TAMs) and the trastuzumab resistance. Then, we developed CD40 ×HER2 bsAb, and examined the targeting roles on HER2 and CD40, to overcome the trastuzumab resistance without systemic toxicity.

**Results:**

We found the level of CCL2 expression in HER2-postive GC was correlated with infiltration of TAMs, polarization status of infiltrated TAMs, trastuzumab resistance and survival outcomes of GC patients. On exposure to CCL2, TAMs decreased the M1-like phenotype, thereby eliciting the trastuzumab resistance. CCL2 activated the transcription of ZC3H12A, which increased K63-linked deubiquitination and K48-linked auto-ubiquitination of TRAF6/3 to inactivate NF-κB signaling in TAMs. CD40 ×HER2 bsAb, which targeted the CD40 to restore the ubiquitination level of TRAF6/3, increased the M1-like phenotypic transformation of TAMs, and overcame trastuzumab resistance without immune-related adversary effects (irAEs).

**Conclusions:**

We revealed a novel mechanism of trastuzumab resistance in HER2-positive GC via the CCL2-ZC3H12A-TRAF6/3 signaling axis, and presented a CD40 ×HER2 bsAb which showed great antitumor efficacy with few irAEs.

**WHAT IS ALREADY KNOWN ON THIS TOPIC**

- Despite the therapeutic success of trastuzumab for HER2-positive gastric cancer (GC), innate or acquired resistance to trastuzumab was still one of the most important causes for treatment failure.
- Overcoming the resistance to trastuzumab remains a critical challenge in patients with HER2-positive GC.

**WHAT THIS STUDY ADDS**

- Our study aimed to investigate the roles of tumor-derived CCL2 on trastuzumab resistance and overcome the resistance by treatment with the anti-CD40-scFv-linked anti-HER2 (CD40×HER2) bispecific antibody (bsAb).

**HOW THIS STUDY MIGHT AFFECT RESEARCH, PRACTICE OR POLICY**

- The finding of CCL2-induced trastuzumab resistance contributes to understanding trastuzumab resistance mechanisms in HER2-positive GC.
- The exploration of molecular mechanism and primary function verification of CD40×HER2 bsAb can offer the evidence for clinical translation and use in the treatment of HER2-positive GC patients.

## Background

Gastric cancer (GC) is a complex and heterogeneous disease that is caused by various genetic, environmental, and host factors. During neoplasia, the interaction network between cancer cells and the tumor microenvironment (TME) creates soil conducive to tumor growth.^1^ Tumor-targeted therapy and immunotherapy have emerged as major therapeutic modalities in oncology. In recent years, the high-throughput technologies, including next-generation sequencing assays, have demonstrated significant progress in identifying powerful diagnostic, prognostic, and therapeutic biomarkers and in the discovery of molecular subtypes of GC.^2^ However, only a few biomarkers have been translated into the clinical trial phase, and fewer molecular-targeted agents have significantly improved outcomes in patients with GC.^3^

HER2 overexpression or gene amplification occurs in approximately 10%–15% of patients with GC.^4^ In 2010, the phase III ToGA study demonstrated that patients with HER2 overexpressing GC got a survival benefit from treatment with the anti-HER2 recombinant humanized monoclonal antibody, trastuzumab.^5^ With the success of the ToGA study, trastuzumab was recommended as the first-line treatment in combination with chemotherapy in patients with HER2-positive GC. Despite the therapeutic success of trastuzumab in HER2-positive GC, innate or acquired resistance to trastuzumab remains one of the most important causes of treatment failure. Overcoming trastuzumab resistance remains a critical challenge for patients with HER2-positive GC. Several potential mechanisms of the trastuzumab resistance have been proposed: (1) HER2 heterogeneity, (2) loss of HER2 positivity/acquired HER2 mutations, (3) HER2 heterodimers, (4) altered intracellular signaling, and (5) the tumor immune microenvironment.^6^ Among these mechanisms, the tumor immune microenvironment is crucial for regulating the antitumor efficacy of trastuzumab. Accumulating evidence indicates that the antitumor activity of trastuzumab-induced antibody-dependent cellular cytotoxicity and complement-dependent cytotoxicity requires the engagement of immune effector cells, including CD8^+^ T cells and macrophages.^7–9^ Recently, immunotherapy has made a breakthrough in cancer treatment. Tumor-associated macrophages (TAMs) are identified as the critical players in crosstalk between cancer cells and their microenvironment.^10^ However, the mechanism of trastuzumab resistance induced by the crosstalk between GC cells and TAMs has not been understood.

Chemokine (C-C motif) ligand 2 (CCL2), also known as macrophage chemoattractant protein 1 (MCP1), is a well-known chemokine that modulates the infiltration and recruitment of monocytes/macrophages through the combination with CCR2. Recent studies have reported that CCL2 plays a role in shaping the TME and influencing cancer progression and even drug resistance, due to its effect on TAM polarization.^11 12^ However, there was also a controversy as to whether CCL2 contributed to educating the macrophages to the M2-like phenotype or M1-like phenotype, and that may differ between diseases and cancer types.^13–15^ In most types of cancers, such as breast^16^ and esophageal cancers,^17^ CCL2 induces TAMs to undergo M2-like phenotypic transformation. However, the involvement of CCL2 and TAMs in HER2-positive GC has not been investigated.

Bispecific antibodies (bsAbs) can simultaneously bind to two different targets, which can have the compound and synergistic effects in cancer immunotherapy.^18^ In our previous study,^19^ we designed an anti-CD40-scFv-linked anti-HER2 (CD40 ×HER2) bsAb that specifically agitates CD40 signaling and inhibits the dimerization of HER2. Although, our previous study showed the stimulating effect of CD40 ×HER2 bsAbs on dendritic cells and the antitumor activity in HER2 overexpressing T6-17 cells,^19 20^ the function and mechanism of CD40 ×HER2 bsAbs on HER2-positive GC cells and TAMs are still not completely understood.

In this study, we revealed the roles of tumor cell-derived CCL2 on TAMs polarization and the resistance to trastuzumab in HER2-positive GC. Further, we investigated the antitumor efficacy and TAMs costimulatory activity of the CD40 ×HER2 bsAb, thereby overcoming the resistance to trastuzumab in HER2-positive GC.

## Methods

### Patients and tissue samples

The tissue specimens used in this study were composed of 33 HER2-positive and 40 HER2-negative tumor tissues, which were all derived from patients with GC receiving curative gastrectomy in department of general surgery in Tianjin Medical University General Hospital (Tianjin, China) between January 2016 and December 2020. All these enrolled patients did not receive neoadjuvant therapy before gastrectomy and their clinicopathological characteristics are summarized in online supplemental table 1.

10.1136/jitc-2022-005063.supp9 Supplementary data

### RNA-sequencing and analysis

Three HER2-positive and three HER2-negative GC tissues were performed the RNA-sequencing analysis. RNA-sequencing service was provided by CloudSeq Biotech (Shanghai, China). Transcriptome high throughput sequencing and subsequent bioinformatics analysis were all done by Cloud-Seq Biotech (Shanghai, China). Briefly, total RNA was used for removing the rRNAs using Ribo-Zero rRNA Removal Kits (Illumina, USA) following the manufacturer’s instructions. RNA libraries were constructed by using rRNA-depleted RNAs with TruSeq Stranded Total RNA Library Prep Kit (Illumina, USA). The 10 pM libraries were denatured as single-stranded DNA molecules, captured on Illumina flow cells, amplified in situ as clusters and finally sequenced for 150 cycles on Illumina HiSeq Sequencer according to the manufacturer’s instructions.

### Preparation and purification of CD40×HER2 bsAb

The expression and purification of CD40 ×HER2 bsAb was described in the previous study.^19^ Briefly, pET28a (+)-CD40×HER2 bsAb plasmid was transformed into *Escherichia coli* Rosetta (DE3). The *E. coli* cells were inoculated in LB medium and were grown at 37℃ until an A600=0.8. Then, Isopropyl-b-D-thiogalactopyranoside was added to promote The *E. coli* cells to express CD40 ×HER2 bsAb for 4 hour. Next, the CD40 ×HER2 bsAb protein was purified through nickel-nitrilotriacetic acid column and the endotoxin was removed Endotoxin Removal Resin (Thermo Fisher Scientific, USA).

### Ubiquitination assay

The ubiquitination assay was described in a previous study.^21^ In this study, we transfected empty vector, pCDNA3.1-UB-K63-HA (human UB, all lysine mutated to arginine, except for K63), pCDNA3.1-UB-K48-HA (human UB, all lysine mutated to arginine, except for K48), pCDNA3.1-TRAF6-Flag, pCDNA3.1-TRAF3-Flag, pCDNA3.1-ZC3H12A-Myc or pCDNA3.1-CD40-His plasmid into HEK-293T cells. After transfection for 42 hours, HEK-293T cells were incubated with MG132 (10 µM) for 6 hours. The proteins were analyzed by the coimmunoprecipitation assay.

### In vivo tumor model

The NCI-N87 cells (5×10^5^ cells in 0.1 mL PBS) stably transfected with pLVX-CCL2 plasmid or pLVX empty vector were injected subcutaneously into the right dorsal flanks of 5-week-old male Bal b/c nude mice. Seven days after injection, the tumor models were confirmed and the treatment were started. The different treatment groups included: (1) NCI-N87-vector cells with the trastuzumab (200 µg/kg), (2) NCI-N87-CCL2 cells with the trastuzumab (200 µg/kg), (3) NCI-N87-CCL2 cells with the trastuzumab (200 µg/kg) and agonistic anti-CD40 mAb (200 µg/kg, BioXCell, USA), (4) NCI-N87-CCL2 cells with CD40 ×HER2 bsAb (200 µg/kg), respectively (n=5). The ongoing treatment was injected intraperitoneally every 3 days until day 31 and tumor volume was also measured.

After last treatment, these mice were sacrificed. The tissues, including tumor tissues, spleen and liver, were surgically harvested. Part of tumor tissue was prepared into single-cell suspensions analyzing the TAMs by flow cytometry. And the formalin-fixed tumor tissues were performed the immunohistochemistry (IHC) staining for caspase 3 to analyze the cell apoptosis in tumor tissues. For toxicity studies, the volume and weight of spleen and liver tissues were measured. And the H&E and IHC staining were preformed to analyze the immune cell population.

The investigator was blinded to the treatment groups during estimating the study. Before injecting tumor cells into mice or sacrificing them, we administered tribromoethanol (350 mg/kg) to the mice by intraperitoneal injection.

Other experimental and statistical methods are available in online supplemental methods. The primers and antibodies used in this study were listed in the online supplemental table 2 and online supplemental table 3, respectively.

10.1136/jitc-2022-005063.supp1 Supplementary data

10.1136/jitc-2022-005063.supp10 Supplementary data

10.1136/jitc-2022-005063.supp11 Supplementary data

## Results

### Tumor cell-derived CCL2 is correlated with polarization of TAMs, trastuzumab resistance and poor prognosis and the in HER2-positive GC patients

**Figure 1:**
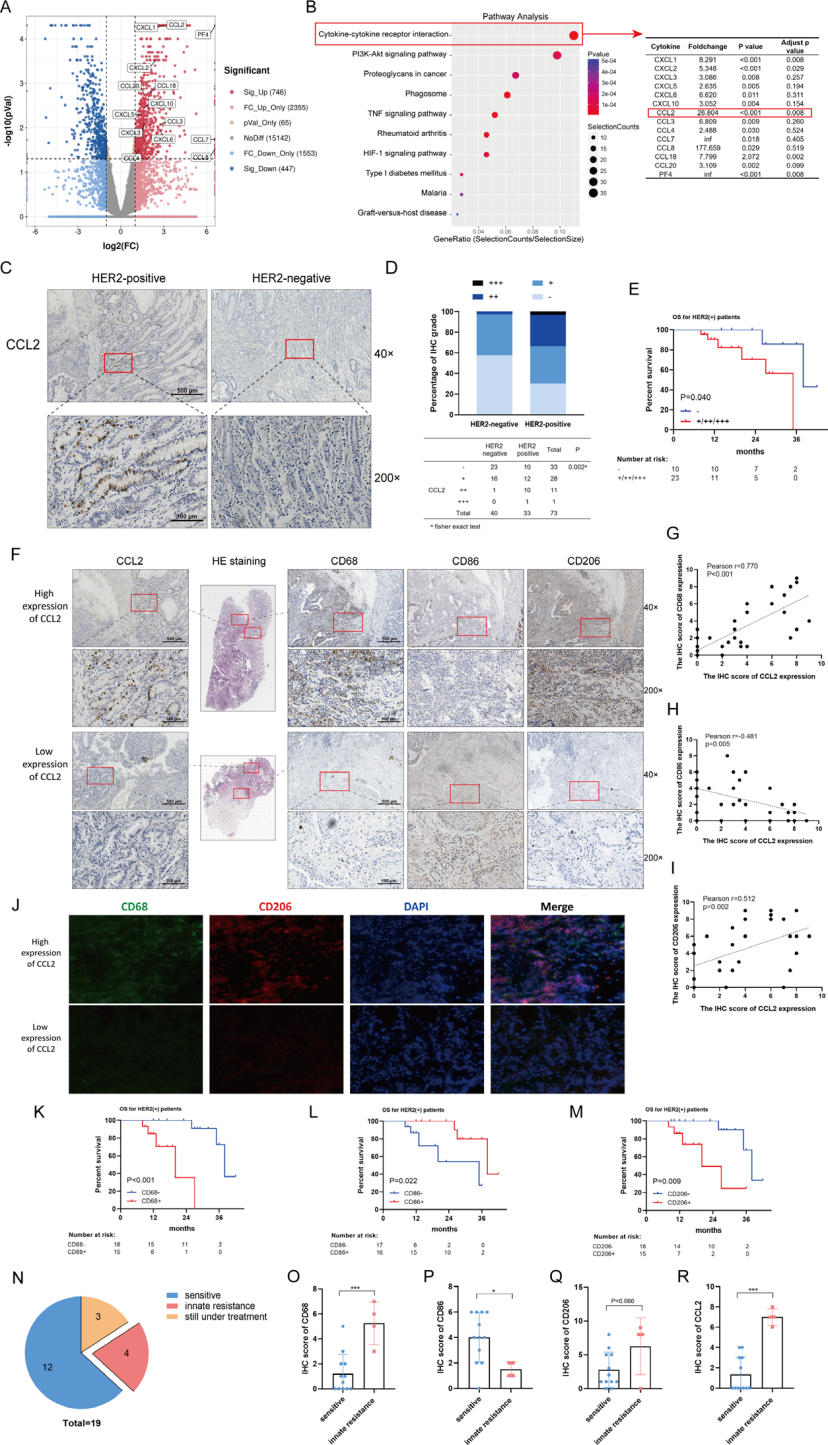
Tumor cell-derived CCL2 is correlated with the polarization of TAMs, the trastuzumab resistance and the poor prognosis in HER2-positive GC patients. (A) The volcano plot was generated from mRNA sequencing analysis of HER2-negative and HER2-positive GC tissues. (B) Gene pathway analysis was performed and the candidate chemokine genes were shown. (C) IHC staining for CCL2 in GC tissues were shown. (D) The different IHC grades of CCL2 expression in HER2-negative and HER2-positive GC tissues was presented. (E) 33 HER2-positive patients were stratified into negative (−) and positive (+/++/+++) groups according to IHC staining for CCL2. Kaplan-Meier analysis of os in HER2-positive GC patients was performed based on CCL2 levels. (F) H&E staining and IHC staining for CCL2, CD68, CD86 and CD206 were shown to present the infiltrating TAMs in human HER2-positive GC tissues. (G–I) Pearson correlation analysis of IHC scores of CCL2 and CD68 (G), CCL2 and CD86 (H), as well as CCL2 and CD206 (I). (J) The immunofluorescence double staining for CD68 and CD206 was shown in human HER2-positive GC tissues. (K–M) 33 HER2-positive patients were categorized into negative (−) and positive (+) groups, based on the median point of IHC score of CD68, CD86 or CD206. Survival analysis of CD68 (K), CD86 (L) and CD206 (M) was showed. (N) The distribution of the trastuzumab resistance in HER2-positive GC patients. (O–R) The different IHC scores of CD68 (O), CD86 (P), CD206 (Q), and CCL2 (R) between the trastuzumab-sensitive and trastuzumab-resistance tissues were presented. scale bar, ×40, 500 µm; ×200, 100 µm. *P<0.05, ***p<0.001. GC, gastric cancer; IHC, immunohistochemistry; OS, overall survival.

To systematically evaluate the gene expression in HER2-positive GC tissues, we employed the genome-wide mRNA sequencing and analyzed mRNA profile in the HER2-negative and the HER2-positive GC tissues. Among the 20 308 genes screened by RNAseq, 1193 genes showed increases/decreased greater than twofold with p<0.05 (figure 1A). The report revealed the cytokine-cytokine receptor interaction pathway involving 14 cytokines markedly upregulated in HER2-positive GC tissues. One of the significantly upregulated genes, CCL2 which involved in monocyte chemotaxis, might play the crucial roles in HER2-positive GC tissues (figure 1B). To confirm CCL2 increasing in the HER2-positive GC tissues, a CCL2 antibody was used to stain 40 HER2-negative and 33 HER2-positive tumor tissues from GC patients (figure 1C). The IHC grade of CCL2 in HER2-positive GC tissues was significantly increased compared with that in HER2-negative tumor tissues (figure 1D).

Next, 33 patients with HER2-positive GC in our center were categorized into CCL2-negative/positive group, based on IHC grade of CCL2. There might be a correlation between CCL2 expression and overall survival (OS) of HER2-positive GC patients (p=0.040), shown in figure 1E. We further examined the corelationship between CCL2 expression and prognosis of HER2-positive GC patients from Gene Expression Omnibus using Kaplan-Meier Plotter tools.^22^ The results showed HER2-positive GC patients with higher CCL2 expression had a poorer OS (p<0.001), first progression (p=0.009) and postprogression survival (PPS) (p=0.008) (online supplemental figure 1A–C). In addition, the survival analysis of CCL2 expression in HER2-negative GC patients (online supplemental figure 1D–F) and entire cohort of GC patients (online supplemental figure 1G–I) was also shown, which verified the negative effect of CCL2 on prognosis of GC patients, especially for HER2-positive GC patients.

10.1136/jitc-2022-005063.supp2 Supplementary data

Multiple studies have shown that CCL2 might play crucial roles in monocyte chemotaxis and even the polarization of macrophage.^23 24^ To determine the correlation between the expression of CCL2 and polarization of TAMs, we examined the CCL2 expression and TAMs biomarkers (Total: CD68, M1-like: CD86, M2-like: CD206) in GC tissues from HER2-positive patients (figure 1F). The IHC results showed a significant correlation between the levels of CCL2 and CD68 (Pearson r=0.770, p<0.001; figure 1G), and those of CCL2 and CD86 (Pearson r=−0.481, p=0.005; figure 1H), as well as those of CCL2 and CD206 (Pearson r=0.512, p=0.002; figure 1I). The immunofluorescence double staining for CD68 and CD206 was shown in figure 1J, which also presented a positive correlation between the CCL2 expression and infiltrated TAMs in HER2-positive GC tissues. In addition, the survival analysis of TAMs biomarkers in HER2-positive GC patients were also shown in the figure 1K–M. The higher expression of CD68 (p<0.001) or CD206 (p=0.009) might lead to the poorer survival outcomes in HER2-positive GC patients, while the lower expression of CD86 (p=0.022) might show a negative effect on prognosis.

Considering that the tumor killing induced by trastuzumab required engagement of immune effector cells, including macrophages, we further examined a relationship between the trastuzumab resistant and TAMs. In this study, 19 (19/33) patients received treatment with trastuzumab plus chemotherapy (figure 1N). Among these patients, 10 (10/19) patients receive the XELOX chemotherapy, 4 (4/19) patients received the SOX chemotherapy, and 5 (5/19) received the S-1 or 5-fluorouracil chemotherapy. After treatment with trastuzumab, 4 cases had progressed within 3 months of trastuzumab therapy (PFS ≤3 m, innate resistance), while the other 12 cases showed the sensitive to trastuzumab within 3 months of trastuzumab (PFS >3 m). The IHC results showed the higher expression of CD68 (p<0.001), CCL2 (p<0.001) and CD206 (p=0.066, a trend with no significance), as well as the lower expression of CD86 (p=0.027) in the innate-resistance patients (figure 1O–R), which indicated that abundant TAMs and the polarization phenotype of TAMs (especially for the reduction of M1-like phenotype) induced by CCL2 were the major barriers for trastuzumab therapy.

These above results indicated that CCL2 could heighten the transition M1-like toward M2-like of TAMs, promoted trastuzumab resistance and caused a poor prognosis of HER2-positive GC patients.

### Tumor cell-derived CCL2 induced trastuzumab resistance and decreased M1-like phenotype of TAMs

**Figure 2:**
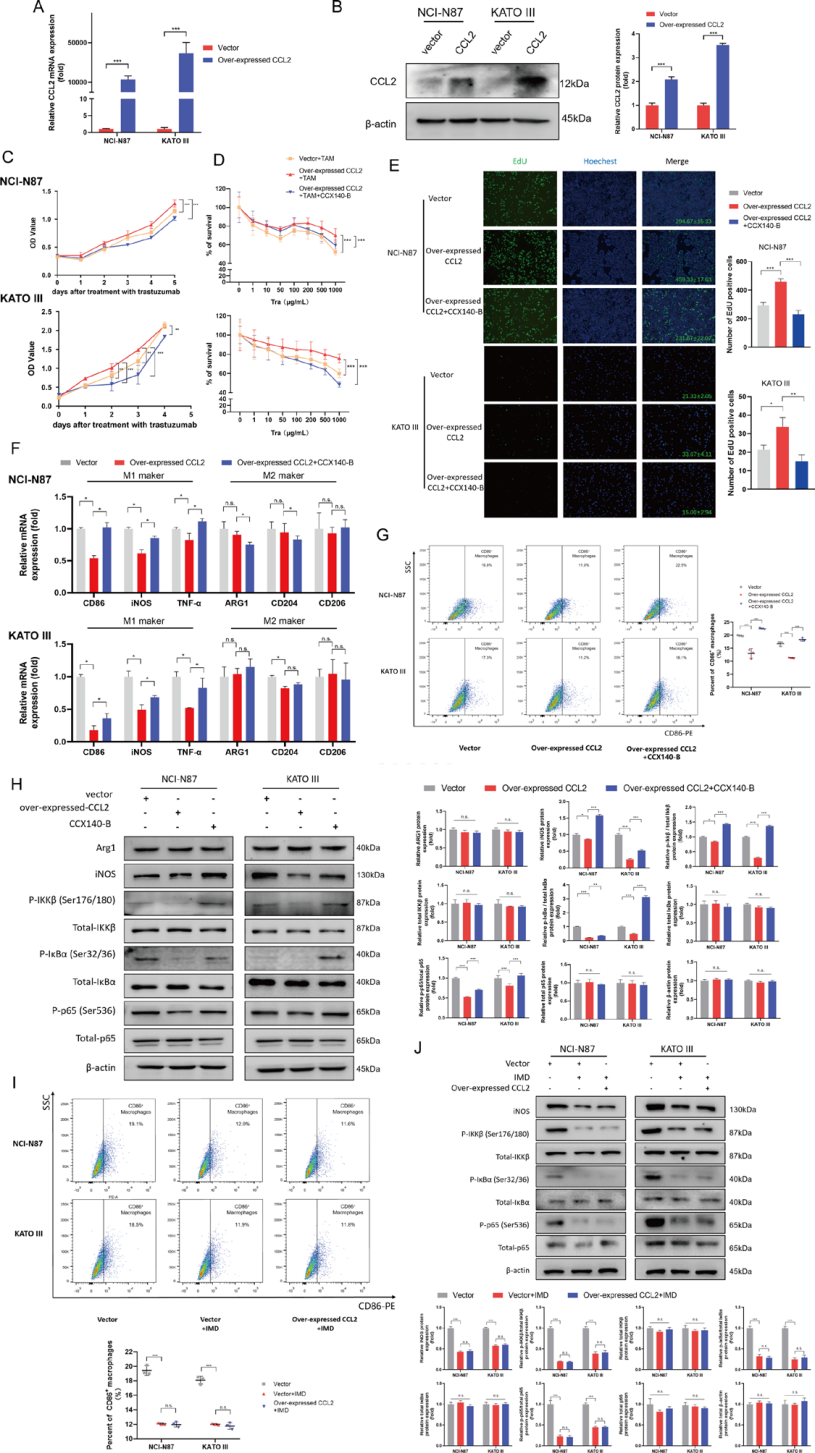
Tumor cell-derived CCL2 decreased the M1-like phenotype of TAMs by via inhibiting NF-κB signaling, therefore eliciting trastuzumab resistance. (A, B) the NCI-N87 and Kato III cell lines were stably transfected with control vector or CCL2 overexpressing plasmid. The expression of CCL2 was detected by PCR (A) and Western blotting (B). (C, D) the stable control (vector +TAM group) and CCL2 overexpressing (overexpressed CCL2 +TAM group) NCI-N87 and Kato III cells were cocultured with TAMs at a ratio 10:1. then, the stable CCL2 overexpressing NCI-N87 and Kato cells cocultured with TAMs, were incubated with CCX140-B to blocked CCL2-CCR2 signaling (overexpressed CCL2 +TAM+CCX140-B group). The mixed cells were incubated with the 50 µg/mL trastuzumab and CCK8 assay was performed to evaluate the drug sensitivity (C). The mixed cells were treated with the trastuzumab at gradient concentrations of from 0 to 1000 µg/mL and CCK8 assay was applied to measure the IC_50_ (D) (n=4). (E–H) TAMs were cocultured with the stable control (vector group) and CCL2 overexpressing (overexpressed CCL2 group) gastric cancer cells in a non-contact coculture Transwell system. Then, the non-contact coculture system of CCL2 overexpressing gastric cancer cells and TAMs were treated with CCX140-B to blocked CCL2-CCR2 signaling (overexpressed CCL2 +CCX140 B group). EdU assay (E) was performed to present the live tumor cells under the treatment with 50 µg/mL trastuzumab (n=3). qPCR (F) and flow cytometry (G) were performed to analyze the polariton phenotype of TAMs (n=3) and Western blotting (H) was performed to analyze the NF-κB signaling pathway. (I, J) TAMs were cocultured with the stable control (vector group) and CCL2 overexpressing gastric cancer cells in a non-contact coculture Transwell system. then, IMD 0354 (IMD, 2 µM) was adopted to block the NF-κB signaling in the TAMs, which were cocultured with stable control (vector +IMD group) or CCL2 overexpressing (overexpressed CCL2 +IMD) gastric cancer cells. The flow cytometry (I) was performed to analyze the polariton phenotype of TAMs (n=3), and Western blotting (J) was performed to analyze the NF-κB signaling pathway. Results were shown as mean±SD. IMD (IMD 0354, an inhibitor of IKKβ). *P<0.05, **p<0.01, ***p<0.001. ns, not significant.

We examined the protumor effect of tumor cell-derived CCL2 in vitro. The human CCL2 gene was stably transfected into HER2-positive GC cell lines, NCI-N87 and KATO III, to increase the CCL2 expression. The stable cell lines with high CCL2 expression were established, detected by qPCR and western blot method (figure 2A, B). Unexpectedly, the CCL2 had no significant effects on the proliferation or the trastuzumab resistance of NCI-N87 and KATO III cells when culturing tumor cells alone (online supplemental figure 2A, B), suggesting the tumor cell-derived CCL2 had no direct effect to promote the tumor progression via an autocrine pathway.

10.1136/jitc-2022-005063.supp3 Supplementary data

Considering the crucial roles of CCL2 on TAMs and the crosstalk between TAMs and GC cells in TME, we examined whether tumor cell-derived CCL2 promoted tumor growth in HER2-positive GC tissues by a paracrine TAM-dependent manner. Therefore, we adopted an in vitro model of TAMs. The human monocyte cell line THP1 was induced into TAMs (online supplemental figure 2C–E), and then cocultured with NCI-N87 and KATO III cells. As shown in figure 2C, D, CCL2 overexpression significantly protected the tumor cells from trastuzumab treatment, elevated the half inhibitory concentration (IC_50_) of trastuzumab and promoted the resistance in NCI-N87 and KATO III cells via a paracrine effect on TAMs. CCX140-B (20 nM, MedChemExpress, USA) treatment, as a specific CCR2 inhibitor, efficiently blocked CCL2-CCR2 signaling confirming the efficacy of CCL2 for TAMs. These results were also verified by the EdU assay (figure 2E). Next, we further investigated the potential mechanism of CCL2 for TAMs. PCR and flowcytometry were performed to analyze the polarization state of TAMs, which was cocultured with NCI-N87 and KATO III cells. As shown in the figure 2F, CCL2 decreased mRNA levels of CD86, iNOS and TNF-α (biomarkers of M1-like phenotype), but caused slight changes on the mRNA levels of ARG1, CD204 and CD206 (biomarkers of M2-like phenotype), detected by qPCR. Moreover, based on the flowcytometry analysis, CCL2 down-regulated the CD86^+^ TAMs without influence on CD206^+^ TAMs in vitro (figure 2G and online supplemental figure 3A). These results were verified by revealing the function of recombinant CCL2 (rCCL2) on macrophages. As shown in online supplemental figure 4A, B, rCCL2 could also downregulate CD86^+^ macrophages from LPS+IFN-γ treatment, indicating that rCCL2 inhibited the M1 phenotype transition, instead of M2 phenotype.

10.1136/jitc-2022-005063.supp4 Supplementary data

10.1136/jitc-2022-005063.supp5 Supplementary data

Because the NF-κB signaling pathway plays essential roles in inflammation and macrophage polarization, we next examined whether CCL2 decreased M1-like phenotype of TAMs by NF-κB signaling pathway. Base on the western blot results, the TAMs which were cocultured with CCL2-overexpression GC cells showed a lower expression of iNOS, p-IKKβ, p-IκBα and p-p65, suggesting that CCL2 might decrease M1-like phenotype of TAMs via inhibiting NF-κB signaling (figure 2H). Then, we adopted IMD 0354 (IMD, Selleck, Houston, USA), an inhibitor of IKKβ, to block the NF-κB signaling in the TAMs. After blocking the NF-κB pathway with IMD (2 µM) treatment, the results of flowcytometry and western blot showed that the roles of CCL2 on macrophage polarization and NF-κB signaling were significantly inhibited (figure 2I, J).

All these results suggested CCL2 effectively decreased M1-like phenotype of TAMs by NF-κB signaling in vitro, to protect the HER2-positive GC cells from trastuzumab treatment.

### Tumor cell-derived CCL2 enhanced the expression of ZC3H12A decreasing M1-like phenotype of TAMs

**Figure 3:**
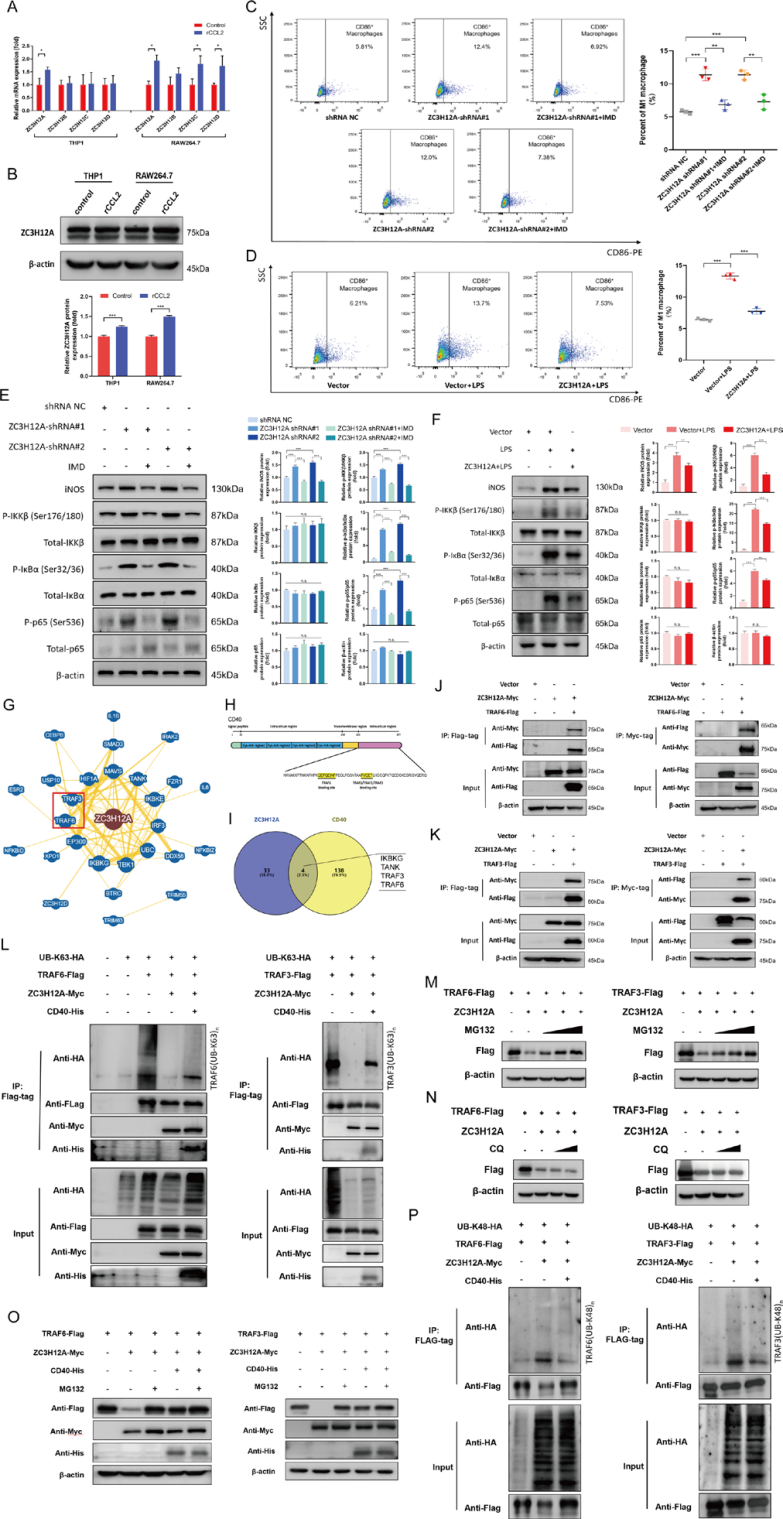
The increase of K63-linked deubiquitination and K48-linked ubiquitination of TRAF6/3 induced by ZC3H12A was recovered by CD40. (A, B) Overexpressing ZC3H12A induced by rCCL2 in THP1 and RAW264.7 Cells was detected by qPCR (A) and Western blotting (B). (C, D) IMD 0354 (IMD) was adopted to block the NF-κB signaling in RAW264.7 cells. Flow cytometry were performed to analyze the polariton phenotype in RAW264.7 cells in which ZC3H12A expression was knocked down (C) or upregulated (D). (E, F) Western blotting was performed to analyze the NF-κB signaling pathway in the ZC3H12A knockdown (E) or overexpressing (F) RAW264.7 cells. (G) Protein interaction network analysis for ZC3H12A was performed using the BioGRID online tools. (H) Structural pattern diagram of CD40 and the binding sites to TRAF6/3 were shown. (I) Venn diagram was presented to show the common binding proteins of ZC3H12A and CD40, based on the the BioGRID online tools. (J, K) ZC3H12A-Myc plasmid or control vector was cotransfected transiently with the TRAF6-Flag (J), TRAF3-Flag (K) plasmid or control vector into HEK-293T cells. Protein lysate was coimmunoprecipitated with Flag-tag (left) and Myc-tag (right) and measured through Western blotting method. (L) HEK-293T cells were transfected with UB-K63-HA, ZC3H12A-Myc, CD40-His and TRAF6-Flag (left), or TRAF3-Flag (right) plasmids. They have proceeded to IP and immunoblot analysis with anti-FLAG beads and were analyzed with anti-HA AB by immunoblot. (M, N) HEK-293T cells were transfected with ZC3H12A-Myc and TRAF6-Flag (left) or TRAF3-Flag (right). After 42 hours, cells were treated with 10, 25, 50 µM MG132 (M) or 20, 40 µM CQ (N) for 6 hour. The TRAF6-Flag (left) or TRAF3-Flag (right) expression was detected by Western blotting. (O) HEK-293T cells were transfected with ZC3H12A-Myc, CD40-His and TRAF6-Flag (left) or TRAF3-Flag (right) plasmids. After 42 hours, cells were treated with 25 µM MG132. The TRAF6-Flag (left) or TRAF3-Flag (right) expression was detected by Western blotting. (P) HEK-293T cells were transfected with UB-K48-HA, ZC3H12A-Myc, CD40-His and TRAF6-Flag (left), or TRAF3-Flag (right) plasmids. They had proceeded to IP and immunoblot analysis with anti-FLAG beads and were analyzed with anti-HA AB by immunoblot. Results are shown as mean±SD. IMD (IMD 0354, an inhibitor of IKKβ). *P<0.05, **p<0.01, ***p<0.001. ns, not significant.

We further explored the underlying mechanism of CCL2 for TAMs. ZC3H12 family proteins, also known as MCPIP family proteins, were first recognized as the induced expression proteins in monocytes treated with CCL2. Some members of ZC3H12 family proteins, such as ZC3H12A or ZC3H12D were well-known modulators of anti-inflammation, which might be also crucial for TAMs. We examined whether the CCL2 decreased M1-like phenotype of TAMs by enhancing the expression of ZC3H12 family proteins. We performed the qPCR and western blot analysis for ZC3H12 family proteins in TAMs induced by rCCL2. These results demonstrated that rCCL2 enhanced the expression of ZC3H12A in THP1 and RAW264.7 cell lines (figure 3A, B). To uncover the effect of CCL2-ZC3H12A axis on macrophages, we established stable RAW264.7 cell lines in which ZC3H12A expression was up-regulated or knocked down (online supplemental figure 5A–D). As shown in figure 3C, D, down-regulation of ZC3H12A elicited the increase of the CD86^+^ macrophages. Conversely, overexpression of ZC3H12A significantly decreased the CD86^+^ macrophages induced by LPS for 6 hour. Moreover, based on the western blot results, down-regulation of ZC3H12A significantly enhanced the expression of iNOS, p-IKKβ, p-IκBα and p-p65, whereas upregulation of ZC3H12A inactivated the NF-κB signaling in RAW264.7 cells (figure 3E, F). Moreover, as shown in the figure 3C, E, IMD reversed the ZC3H12-induced increase in CD86^+^ macrophages and activation in NF-κB signaling in RAW264.7 cells, which further confirmed the roles of ZC3H12A on NF-κB pathway.

10.1136/jitc-2022-005063.supp6 Supplementary data

### The increase of K63-linked deubiquitination and K48-linked ubiquitination of TRAF6/3 induced by ZC3H12A was recovered by CD40

To determine the mechanism of ZC3H12A, we performed the protein interaction network analysis for ZC3H12A, according to the BioGRID online tools (figure 3G). Among these interacting proteins, TRAF6 and TRAF3 were recognized as the important proteins, which has been reported to play essential roles for activating NF-κB signaling.^25 26^ ZC3H12A, as a deubiquitinating enzyme, might deubiquitinate TRAF6/3 to inhibit the NF-κB signaling pathway.^27^ Conversely, CD40 upregulated the immune response through interacting TRAF family proteins (figure 3H, I).^28^ Thus, we examined whether CD40 protected the TRAF6 and TRAF3 from deubiquitination effect of ZC3H12A. To further assess this hypothesis, ZC3H12A gene and TRAF6 or TRAF3 gene were transfected into HEK-293T cells, and then CO-IP assay was performed. We found ZC3H12A protein interacted with TRAF6 and TRAF3 proteins (figure 3J, K). Subsequently, we used the K63 ubiquitin (all lysine mutated to arginine, except for K63) to test the deubiquitination effects of ZC3H12A and CD40 in HEK-293T cells. As shown in figure 3L, the decreased K63-linked ubiquitination of TRAF6 and TRAF3 induced by ZC3H12A were up-regulated by activating CD40.

Furthermore, we found ZC3H12A significantly reduced the protein levels of TRAF6 and TRAF3 (figure 3J, K). TRAF6 and TRAF3, as E3 ubiquitin ligases, were reported to have potential autoubiquitination to mediate the NF-κB signaling.^29^ Thus, we examined whether ZC3H12A increased TRAF6/3-mediated K48-linked ubiquitination to degrade TRAF6/3 proteins by the proteasome pathway. We tested the protein levels of TRAF6/3 in HEK-293T cells treated with MG132, a proteasomal inhibitor, or chloroquine (CQ), a lysosomal inhibitor. The protein levels of TRAF6 and TRAF3 could be recovered by MG132, but not CQ (figure 3M, N). CD40 significantly increased the stability of TRAF6/3, and protected TRAF6/3 from proteasomal degradation induced by K48-linked ubiquitination (figure 3O). To determine the K48-linked ubiquitination levels of TRAF6 and TRAF3 induced by ZC3H12A, the K48 ubiquitin (all lysine mutated to arginine, except for K48) was used to performed the ubiquitination assay. As shown in figure 3P, ZC3H12A significantly increased the K48-linked ubiquitination of TRAF6 and TRAF3. Conversely, CD40 reduced the K48-linked ubiquitination induced by ZC3H12A.

These results suggested ZC3H12A significantly inhibited NF-κB signaling by promoting K63-linked deubiquitination and K48-linked ubiquitination of TRAF6/3. In contrast, CD40 significantly enhanced K63-linked ubiquitination and reduced the K48-linked ubiquitination of TRAF6/3 reactivating NF-κB signaling. ZC3H12A and CD40 together maintained a K63-linked and K48-linked balance of TRAF6/3, which was critical in NF-κB signaling.

### CD40×HER2 bsAb increased M1-like phenotype of TAMs, and overcame trastuzumab resistance

**Figure 4:**
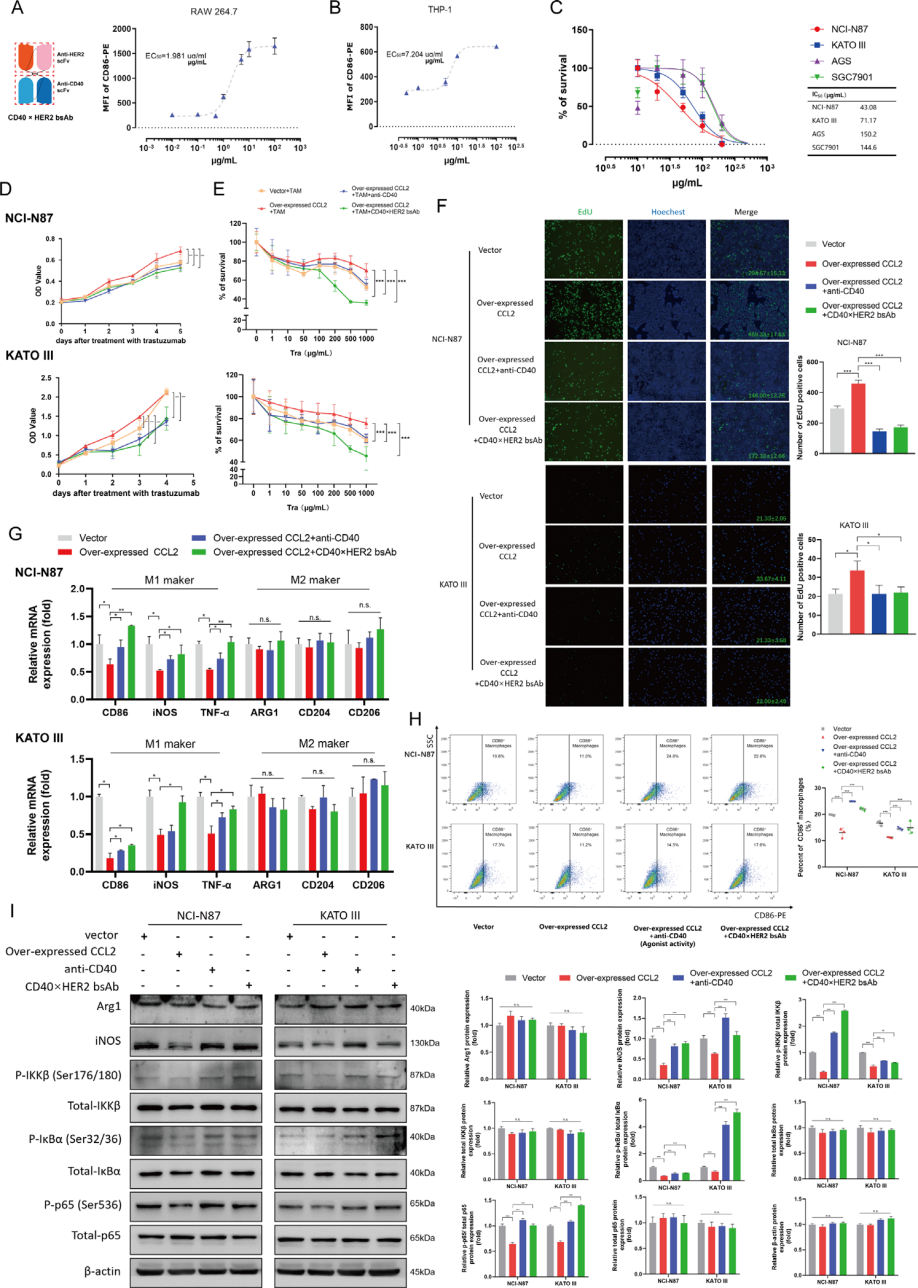
CD40 ×HER2 bsAb increased M1-like phenotype of TAMs, and overcame trastuzumab resistance in vitro. (A, B) Dose-dependent costimulatory activity of CD40 ×HER2 bsAb on RAW264.7 cells (A) and THP1 cells (B) was shown (n=3). (C) Dose-dependent antitumor activity of CD40 ×HER2 bsAb on HER2-negative cell lines (AGS and SGC7901), and HER2-positive cell lines (NCI-N87 and KATO III) (n=5) was presented. (D, E) The stable control (vector +TAM group) and CCL2 overexpressing (overexpressed CCL2 +TAM group) NCI-N87 and KATO III cells were cocultured with TAMs at a ratio 10:1. The CCL2 overexpressing NCI-N87 and KATO III cells cocultured with TAMs, were treated with the agonistic anti-CD40 antibody (overexpressed CCL2 +TAM+anti-CD40) or CD40 ×HER2 bsAb (overexpressed CCL2 +TAM+ CD40×HER2 bsAb). Then, the mixed cells were incubated with the 50 µg/mL trastuzumab and CCK8 assay was performed to evaluate the drug sensitivity (D). The mixed cells were treated with the trastuzumab at gradient concentrations of from 0 to 1000 µg/mL and CCK8 assay was applied to measure the IC_50_(E) (n=4). (F–I) TAMs were cocultured with the stable control (vector group) and CCL2 overexpressing (overexpressed CCL2 group) gastric cancer cellsin a non-contact coculture transwell system. The coculture system of CCL2 overexpressing gastric cancer cells and TAMs was incubated with the agonistic anti-CD40 antibody (overexpressed CCL2 +anti-CD40 group) or CD40 ×HER2 bsAb (overexpressed CCL2 +CD40×HER2 bsAb group) treatment. EdU assay (F) was performed to present the live tumor cells under the treatment with 50 µg/mL trastuzumab (n=3). qPCR (G) and flow cytometry (H) were performed to analyze the polariton phenotype of TAMs with with CD40 ×HER2 bsAb treatment (n=3). And western blotting (I) was performed to analyze the NF-κB signaling pathway. Results were shown as mean±SD. *P<0.05, **p<0.01, ***p<0.001. bsAb, bispecific antibody.

Based on the molecular mechanism, we designed CD40 ×HER2 bsAb, which specifically inhibited the dimerization of HER2 and agitated CD40 signaling. In this study, we first determined the functional characterization of CD40 ×HER2 bsAb in TAMs and HER2-positive GC cells. As shown in figure 4A, B, CD40 ×HER2 bsAb increased the expression of CD86 on RAW264.7 cells and THP1 cells in a dose-dependent manner. Moreover, CD40 ×HER2 bsAb inhibited the cell proliferation of HER2-positive GC cells with lower IC_50_ (NCI-N87, 43.08 µg/mL and KATO III, 71.17 µg/mL, figure 4C). These results indicated the CD40 ×HER2 bsAb had a CD40 targeted macrophage-costimulatory activity and a HER2-mediated antitumor activity.

Base on the above results, we examined whether CD40 ×HER2 bsAb could increase the M1-like phenotype of TAMs overcoming trastuzumab resistance. In an in vitro coculture model, both agonistic anti-CD40 (40 ng/mL, R&D, USA) and CD40 ×HER2 bsAb (20 µg/mL) significantly promoted the cells killing from trastuzumab treatment, declined the IC_50_ of trastuzumab and overcame the trastuzumab resistance (figure 4D, E). EdU staining also verified that treatment with agonistic anti-CD40 Ab or CD40 ×HER2 bsAb inhibited the cell proliferation and overcame the trastuzumab resistance in NCI-N87 and KATO III cells (figure 4F). Next, we further tested the polarization phenotype of TAMs cocultured with HER2-positive GC cells. Treatment with CD40 ×HER2 bsAb increased the mRNA levels of CD86, iNOS and TNF-α in TAMs in qPCR analysis and the frequency of CD86 +TAMs in flowcytometry analysis, whereas CD40 ×HER2 bsAb have a little effect on M2-like TAMs phenotype in vitro (figure 4G, H and online supplemental figure 3B). In addition, we performed western blot assays to analyze the changes on the NF-κB signaling in TAMs, and found treatment with CD40 ×HER2 bsAb enhanced the levels of iNOS, p-IKKβ, p-IκBα and p-p65 in TAMs (figure 4I).

Collectively, all these results suggested CD40 ×HER2 bsAb generated a compound and synergistic antitumor response with HER2 blockade and CD40 targeted costimulatory activity, which increases M1-like phenotype of TAMs and overcomes trastuzumab resistance in HER2-positive GC.

### CD40×HER2 bsAb overcame trastuzumab resistance in the xenograft model

**Figure 5:**
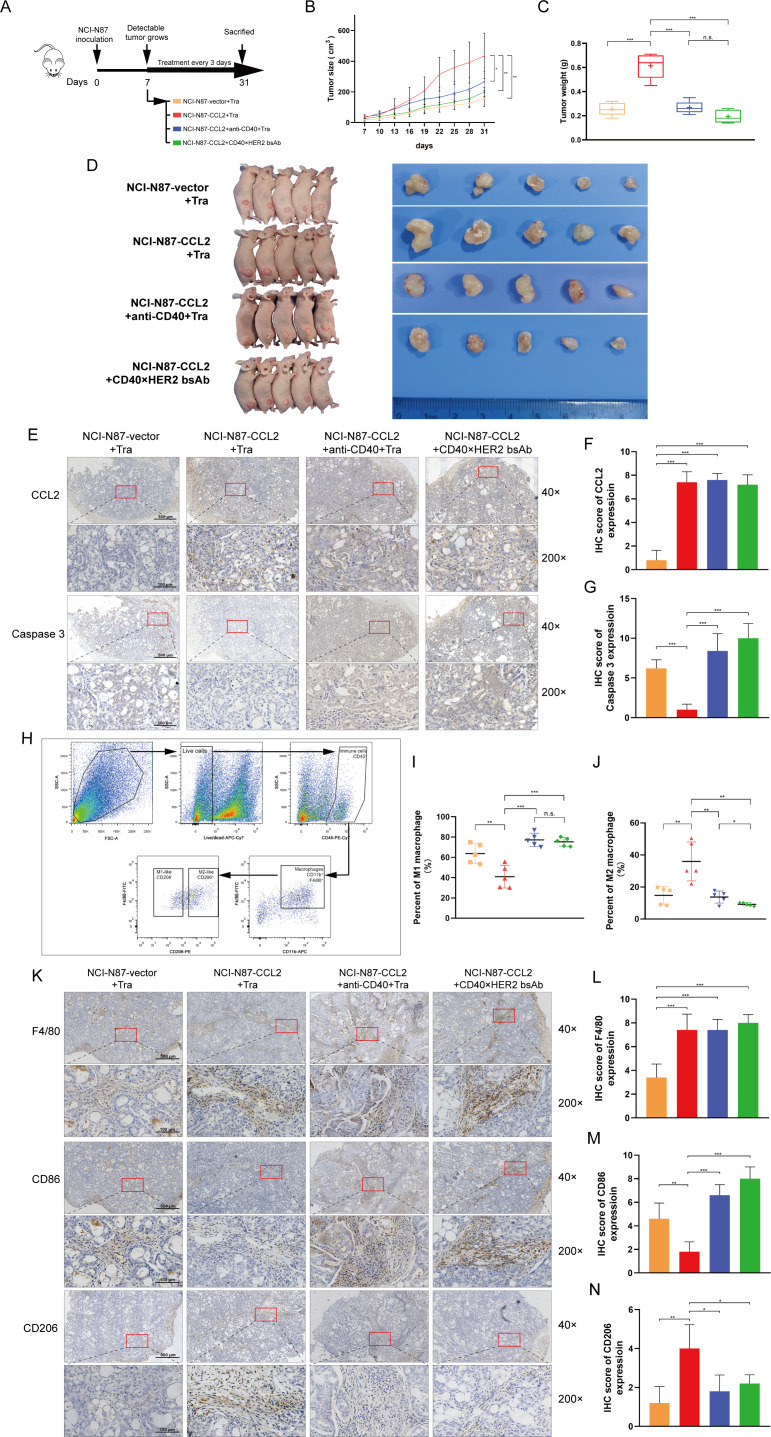
CD40 ×HER2 bsAb overcame trastuzumab resistance in the xenograft model. (A) Experimental scheme of CD40 ×HER2 bsAb treatment in NCI-N87-tumor-bearing Balb/c nude mice. The different treatment groups included: (1) the stable control NCI-N87 cells with the trastuzumab treatment (NCI-N87-vector+Tra group), (2) the stable CCL2 overexpressing NCI-N87 cells with the trastuzumab treatment (NCI-N87-CCL2+Tra group), (3) the stable CCL2 overexpressing NCI-N87 cells with the trastuzumab and agonistic anti-CD40 mAb treatment (NCI-N87-CCL2+anti-CD40+Tra group), (4) the stable CCL2 overexpressing NCI-N87 cells with CD40 ×HER2 bsAb treatment (NCI-N87-CCL2+CD40×HER2 bsAb group). (B–D) Tumor growth curves (B), tumor weight (C) and tumor volume images (D) of each group were shown. (E–G) IHC staining for CCL2 and caspase three was presented (E), and the IHC scores for CCL2 (F) and caspase 3 (G) were statistically analyzed. (H) The gating strategy to identify CD45^+^ CD11b^+^ F4/80^+^ CD206^+/-^ TAMs in mouse tumor tissues. (I, J) The infiltrating CD45^+^ CD11b^+^ F4/80^+^ CD206^-^ (I) or CD45^+^ CD11b^+^ F4/80^+^ CD206^+^ (J) TAMs percentages in the whole cell counts detected by flow cytometry were statistically analyzed. (K–N) IHC staining for F4/80, CD86, and CD206 was presented to show the infiltrating TAMs in mouse tumors (K). IHC scores for F4/80 (L), CD86(M) and CD206 (N) were statistically analyzed. n=5 per group. tra, trastuzumab treatment. Scale bar, ×40, 500 µm; ×200, 100 µm. *P<0.05, **p<0.01, ***p<0.001. bsAb, bispecific antibody; IHC, immunohistochemistry; ns, not significant.

To demonstrate the above in vitro results, an in vivo xenograft model was used. NCI-N87 with the control or CCL2 overexpression were subcutaneously injected into the female Balb/c nude mice. The trastuzumab (200 µg/kg), trastuzumab (200 µg/kg) with agonistic anti-CD40 mAb (200 µg/kg), or CD40 ×HER2 bsAb (200 µg/kg) was injected intraperitoneally at the day 7, and continued every 3 days until day 31 (figure 5A). As shown in the figure 5B–D, CCL2 upregulation resulted in a substantial elevation of tumor volume and weight in mouse model. However, the CCL2-induced elevation of GC tumor volume and weight was greatly decreased with anti-CD40 mAb or CD40 ×HER2 bsAb treatment. Furthermore, the caspase 3 antibody was used to stain the apoptotic cells in GC tissues. The caspase 3 staining was significantly decreased in tumors derived from CCL2-overexpression GC cells, and increased after anti-CD40 mAb or CD40 ×HER2 bsAb treatment (figure 5E–G). These results indicated the CD40 ×HER2 bsAb treatment overcame the trastuzumab resistance induced by CCL2.

Next, we examined whether CCL2-induced trastuzumab resistance which was overcame by CD40 ×HER2 bsAb treatment, was mediated by TAMs. The antibodies for CD45, CD11b, F4/80, and CD206 were used to label the mouse TAMs to perform the flowcytometry. The gating strategy used to identify CD11b^+^F4/80^+^CD206^-^ (M1-like) and CD11b^+^F4/80^+^CD206^+^ (M2-like) TAMs in mouse tumors was shown in figure 5H. As shown in the figure 5I,J, CCL2 significantly decreased the proportion of CD45^+^ CD11b^+^ F4/80^+^ CD206^-^ (M1-like) TAMs and increased the CD45^+^ CD11b^+^ F4/80^+^ CD206^+^ (M2-like) TAMs in mouse tumor tissues. However, anti-CD40 mAb or CD40 ×HER2 bsAb treatment reversed the changes of polarization phenotypes of TAMs induced by CCL2 in mouse GC tissues. To further determine the polarization phenotypes of TAMs in the mouse GC tissues, IHCs for F4/80, CD86 and CD206 were performed. As shown in the figure 5K–N, the IHC results confirmed the polarization phenotypes of TAMs that the down-regulated CD86 staining and up-regulated CD206 staining induced by CCL2 were significantly reversed by treatment with anti-CD40 mAb or CD40 ×HER2 bsAb.

These results indicated that CD40 ×HER2 bsAb could overcome trastuzumab resistance in the xenograft model.

### CD40×HER2 bsAb treatment avoided immune-related adversary effects

**Figure 6:**
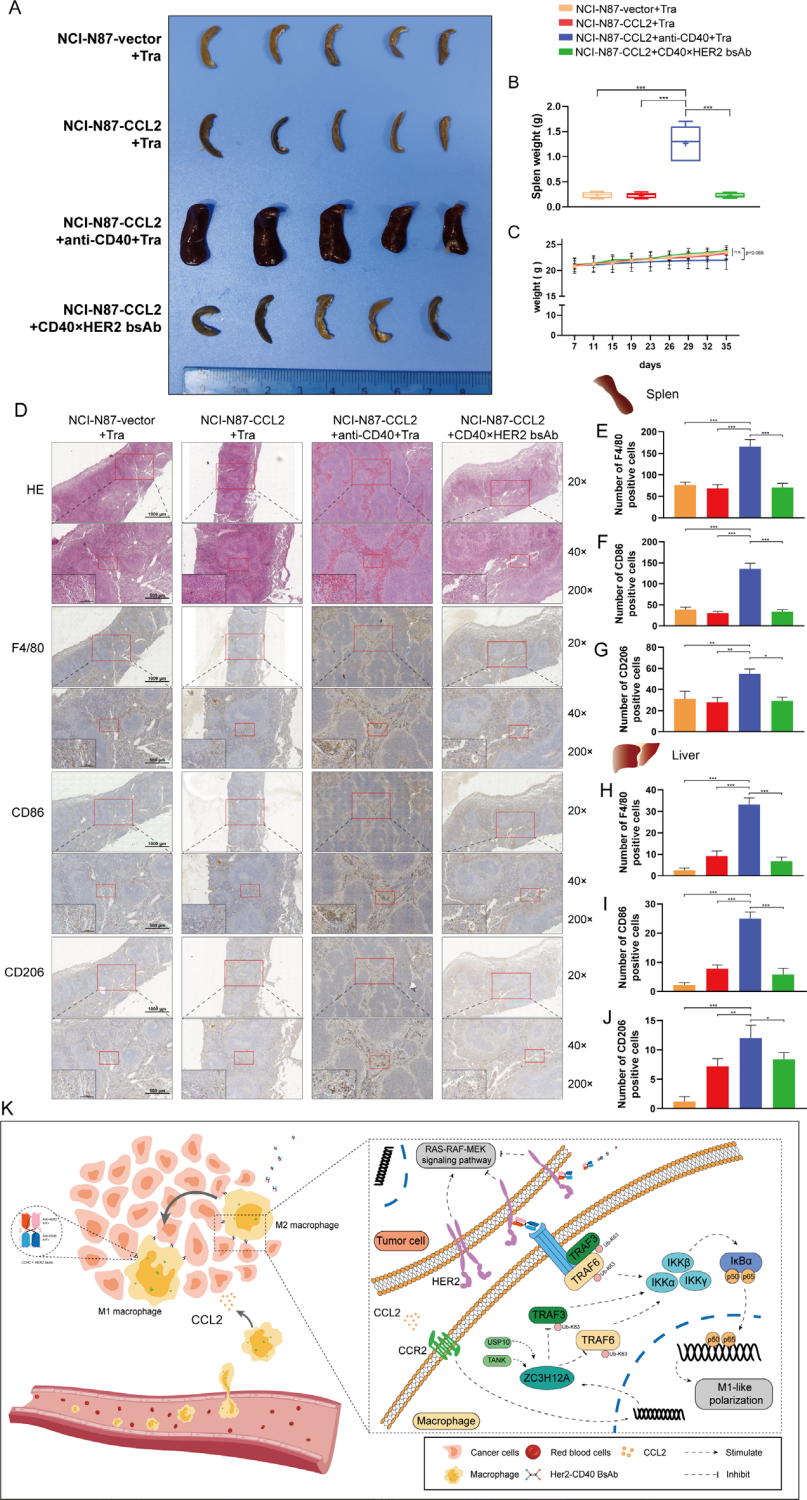
CD40 ×HER2 bsAb treatment avoided irAEs. (A–C) Spleen volume images (A), spleen weight (B) and mouse body weight curves (C) of each group were presented. (D–G) H&E staining and IHC staining for F4/80, CD86, and CD206 were presented to show the infiltrating macrophages in mouse spleen tissues (D). IHC scores for F4/80 (E), CD86 (F) and CD206 (G) were statistically analyzed. (H–J) IHC staining in mouse liver tissues for F4/80, CD86, and CD206 was performed. IHC scores for F4/80 (H), CD86 (I) and CD206 (J) were statistically analyzed in mouse liver tissues. (K) Schematic diagram: Tumor cell-derived CCL2 decreased M1-like phenotype of TAMs via ZC3H12A-TRAF6/3 signaling, whereas CD40 markedly protected the TRAF6/3 from K63-linked deubiquitination, thereby reactivating the NF-κB signaling. CD40 ×HER2 bsAb had the CD40 targeted macrophages costimulatory activity and a HER2-mediated antitumor activity, with absence of irAEs, which overcame trastuzumab resistance in GC. n=5 per group. tra, trastuzumab treatment. Scale bar, ×20, 1000 µm; ×40, 500 µm; ×200, 100 µm. *P<0.05, **p<0.01, ***p<0.001. bsAb, bispecific antibody; GC, gastric cancer; IHC, immunohistochemistry; irAEs, immune-related adversary effects.

To address the toxicity induced by CD40 stimulation by comparing CD40 ×HER2 bsAb and agonistic anti-CD40 mAb, we observed the important immune organs, the liver and spleen. As show in the figure 6A, B, agonistic anti-CD40 mAb treatment induced lymphadenopathy and splenomegaly as determined by volume and weight, while CD40 ×HER2 bsAb did not cause any changes in the shape and conformation of spleen. H&E and IHC staining in spleen tissues for F4/80, CD86 and CD206 showed agonistic anti-CD40 strongly induce an increase in the monocyte population and the frequency of CD86^+^ monocyte, whereas splenic immune cell composition was slightly altered following treatment with CD40 ×HER2 bsAb (figure 6D–G). Next, agonistic anti-CD40 treatment caused enlargement of the liver, and markedly increased infiltration of lymphocytes and CD86^+^ monocytes. However, CD40 ×HER2 bsAb did not cause any change in the infiltration of immune cells into the liver (figure 6H–J and online supplemental figure 6). Apart from this, agonistic anti-CD40 might induce the trend of weight loss (figure 6C). These results suggested CD40 ×HER2 bsAb treatment could avoid immune-related adversary effects (irAEs).

10.1136/jitc-2022-005063.supp7 Supplementary data

In summary, we summarized our findings in a schematic diagram (figure 6K). Our study illustrated the trastuzumab resistance mechanism mediated by a crosstalk between tumor cells and TAMs in the GC microenvironment. Tumor cell-derived CCL2 decreased M1-like phenotype of TAMs via ZC3H12A-TRAF6/3 signaling, whereas CD40 markedly protected the TRAF6/3 from K63-linked deubiquitination, thereby reactivating the NF-κB signaling. To achieve the synergistic effect of antitumor and regulating tumor immunity, CD40 ×HER2 bsAb was developed. Our study showed CD40 ×HER2 bsAb had the CD40 targeted macrophage-costimulatory activity and a HER2-mediated antitumor activity, with absence of irAEs, which overcame trastuzumab resistance in GC.

## Discussion

Although trastuzumab plus chemotherapy was the standard treatment for patients with advanced metastatic HER2-positive GC, the overall response rate was only 47% (complete response, 5%; partial response, 42%), according to the ToGA study.^5^ Considering that the antitumor efficacy of trastuzumab partly relies on the immune response status in the TME, one possible reason was that tumor cells could secrete pro-tumor factors to inhibit the immune response and promote immune escape.^6 30 31^ A previous study identified abundant TAMs and the polarization phenotype of TAMs as the major barriers to trastuzumab therapy in breast cancer.^32^ However, the relationship between TAMs and trastuzumab resistance in GC and its potential mechanisms are largely unclear. In this study, we demonstrated that the overexpression of CCL2 derived from HER2-positive GC cells resulted in a decrease of the M1-like phenotype of TAMs and recruited the M2-like phenotype of TAMs in the TME, thereby promoting trastuzumab resistance.

Although CCL2 is the key chemokine for the recruitment and migration of monocytes/macrophages, CCL2 also played a vital role on TAM polarization. In this study, we demonstrated that CCL2 inhibited the M1-like phenotype of TAMs and slightly changed M2-like phenotypic transformation in vitro, whereas in vivo, CCL2 inhibited M1-like phenotypic transformation with a higher ratio of the M2-like phenotype of TAMs in GC tissues. Considering the critical roles of CCL2 on macrophage migration, the different results between in vitro and in vivo experiments might have been due to the fact that CCL2 might recruit more TAMs in the TME, leading to a high ratio of M2-like phenotypic TAMs in tumor tissues. However, CCL2 indeed shaped the macrophage polarization in GC tissues. The underlying mechanism of CCL2 on macrophage polarization remains unclear. In this study, we found that the inhibitory effect of CCL2 on the M1-like phenotypic transformation was mediated by ZC3H12A. ZC3H12A, also known as Regnase-1 and MCPIP1, is recognized as a regulator of RNA metabolic processes and a deubiquitinating enzyme that regulates the protein levels.^33^ In this study, TRAF6 and TRAF3 were identified as the key substrates of ZC3H12A that regulate the NF-κB signaling pathway. ZC3H12A elicited a reduction in K63-linked ubiquitination and an increase in K48-linked ubiquitination of TRAF6/3, leading to the attenuation of immune recognition and killing of tumor cells. TRAF6 and TRAF3 are critical for signal transduction in the NF-κB signaling pathway induced by LPS, IL-1β, and TNF-α.^34^ Moreover, in our previous study, we found that CD40 activates the immune response for organ transplantations and tumor cells.^20 35^ In a study by Pullen *et al*,^36^ CD40 was shown to bind to TRAF6 and TRAF3 proteins via its cytoplasmic domain, suggesting that CD40 activated the NF-κB signaling pathway in a TRAFs-dependent manner. In this study, we found CD40 significantly enhanced K63-linked ubiquitination and reduced K48-linked ubiquitination of TRAF6/3, thereby reactivating NF-κB signaling.

To date, several agonistic anti-CD40 antibodies, such as dacetuzumab (SGN-40), CP-870,893, and ChiLob7/4, have been developed and evaluated in early-phase clinical trials.^37–39^ However, agonistic anti-CD40 antibodies have not been advanced beyond early clinical trials because of their low efficacy and potential toxicity due to several autoimmune reactions, including cytokine release syndrome, hyper-immune stimulation syndrome, and thromboembolic disease.^40^ Although there are new drug developments and ongoing clinical trials involving agonistic anti-CD40 antibodies (such as NCT04364230/NCT03329950/NCT02482168/NCT03123783/NCT04130854), the universal expression of CD40 in dendritic cells, macrophages, B cells, NK cells, and even platelets is the critical reason for toxicity and adverse events, leading to limitations in clinical use.^41^ In this study, we found that treatment with agonistic anti-CD40 Ab in a mouse model might elicit lymphadenopathy, splenomegaly, hepatomegaly, and even weight loss. To overcome these issues, we presented a CD40 ×HER2 bsAb, which targeted HER2-positive GC cells and agitated CD40 signaling in the TME. This study provided proof that treatment with CD40 ×HER2 bsAbs showed great antitumor efficacy with significantly reduced irAEs. We observed that CD40 ×HER2 bsAb increased the M1-like phenotype of TAMs, suggesting that CD40 ×HER2 bsAb elicited great antitumor immunity, although further investigation on the role of other immune cells in CD40 ×HER2-bsAb-mediated antitumor immunity is needed.

In summary, we revealed a novel mechanism of trastuzumab resistance in HER2-positive GC via the CCL2-ZC3H12A-TRAF6/3 signaling axis. To overcome trastuzumab resistance, we presented a CD40 ×HER2 bsAb, which showed great antitumor efficacy with few irAEs.

10.1136/jitc-2022-005063.supp8 Supplementary data

## Data Availability

Data are available in a public, open access repository. Data are available on reasonable request. The RNA-sequencing data can be obtained from the NCBI Sequence Read Archive (BioProject ID SUB10505668).
