# Supplementary material for: CD40×HER2 bispecific antibody overcomes the CCL2-induced trastuzumab resistance in HER2-positive gastric cancer
Source: J Immunother Cancer. 2022 Jul 15;10(7):e005063. doi: 10.1136/jitc-2022-005063 (PMC9295658; doi:10.1136/jitc-2022-005063)
Supplement: Supplementary data [file jitc-2022-005063supp009.pdf]

Supplementary Table 1. Clinicopathologic features of patients with HER2-positive and HER2-negative GC

Characteristics	HER2-negative N=40	HER2-positive n=33	P value
**Gender**			
Male	30	30	0.077
Female	10	3	
**Age**			
<60	6	8	0.318
≥60	34	25	
**Tumor location**			
Upper third	5	5	0.870
Middle third	11	9	
Lower third	15	14	
More than 2/3 stomach	9	5	
**Tumor size**			
<4cm	14	7	0.195
≥4cm	26	26	
**pT stage**			
pT1	6	3	0.767[a]
pT2	6	3	
pT3	4	3	
pT4a	20	18	
pT4b	4	6	
**pN stge**			
pN0	10	10	0.398
pN1	8	10	
pN2	8	8	
pN3a	9	4	
pN3b	5	1	
**pTNM stage**			
I	8	4	0.467
II	5	7	
III	27	22	
**Histopathological classification**			
High-moderately differentiated adenocarcinoma	24	16	0.325
Low-undifferentiated adenocarcinoma	16	17	
**Borrman type**			
I	2	1	0.554[a]
II	9	4	
III	10	7	

IV	19	21

Note: [a], fisher exact test; [*], P<0.05;
