# Supplementary material for: CD40×HER2 bispecific antibody overcomes the CCL2-induced trastuzumab resistance in HER2-positive gastric cancer
Source: J Immunother Cancer. 2022 Jul 15;10(7):e005063. doi: 10.1136/jitc-2022-005063 (PMC9295658; doi:10.1136/jitc-2022-005063)
Supplement: Supplementary data [file jitc-2022-005063supp001.pdf]

Supplementary Figure Legends：

Supplementary Figure 1. High CCL2 expression was correlated with poorer outcomes in GC patients (A-C) Kaplan–Meier analysis of OS (A), FP(B), PPS(C) in 343 HER2-positve patients from GEO was performed. (D-F) Kaplan–Meier analysis of OS (D), FP (E), PPS (F) in 532 HER2-negative GC patients from GEO was performed. (G-I) Kaplan–Meier analysis of OS (G), FP (H), PPS (I) in entire 875 GC patients from GEO was performed.

Supplementary Figure 2. The induction and identification of macrophage from THP1 cells were shown. (A-B) The tumor cell growth curves were presented to show the autocrine effect of CCL2 in CCL2-overexpressing and control HER2-positive GC cell lines without (A) or with (B) trastuzumab treatment (n=4). (C) Flow chart of inducing TAMs was presented. (D-E) qPCR (D) and flow cytometry (E) were performed to identify the M0 induced from THP1 cells. * $P < 0.05$

Supplementary Figure 3. CCL2 and CD40×HER2 bsAb treatment slightly altered the M2-like phenotype of TAMs. (A-B) TAMs were co-cultured with NCI-N87 and KATO III cells in a non-contact co-culture transwell system. CCL2 (A) and CD40×HER2 bsAb treatment (B) slightly altered the percent of $CD206^{+}$ TAMs (n=3). *** $P < 0.001$.

Supplementary Figure 4. rCCL2 decreased the M1-like polarization phenotype of macrophages *in vitro*. (A) rCCL2 downregulated $CD86^{+}$ macrophages from

LPS+IFN-γ treatment (n=3). (B) rCCL2 didn't cause any change in percent of $CD206^+$ macrophages with IL-4 and IL-13 treatment (n=3). * P < 0.05, *** P < 0.001.

Supplementary Figure 5. ZC3H12A expression was stably up-regulated or knocked down in RAW264.7 cell lines. (A-B) ZC3H12A overexpressing RAW264.7 cell line was stably established. The expression of ZC3H12A in RAW264.7 cells was detected by PCR (A) and western blotting (B). (C-D) ZC3H12A knockdown RAW264.7 cell line was stably established, detected by by PCR (C) and western blotting (D).

Supplementary Figure 6. H&E staining and IHC staining for F4/80, CD86, and CD206 were presented to show the infiltrating macrophages in mouse spleen tissues. Scale bar,20×, 1000μm; 40×, 500μm; 200×, 100μm.

Supplementary Figure 7. Unprocessed images of western blots. Unprocessed images of immunoblots shown in Figures 2-4 and Supplementary Figure 5 are provided.

Supplementary Tables：

Supplementary Table 1. Clinicopathologic features of patients with HER2-positive and HER2-negative GC

Supplementary Table 2. Real-time PCR primers used in this study

Supplementary Table 3. Main antibodies adopted in study

Supplementary methods:

Survival analysis of Gene Expression Omnibus (GEO) data

GEO data of 875 patients with GC were analyzed on the website tool, Kaplan-Meier Plotter (http://kmplot.com/analysis/index.php?p=background) [1]. The normalized RNA expressions of CCL2 were calculated from a ratio of CCL2 and HPRT1.

Cell lines

The cell lines, including two HER2-positive GC cell lines (NCI-N87 and KATO III), a human monocyte cell line (THP1), a mouse macrophage cell line (RAW264.7) as well as a human embryonic kidney cell line (HEK-293T), were all obtained from the American Type Culture Collection (ATCC, Manassas, VA, USA). NCI-N87 and KATO III were cultured in RPMI 1640 medium (Gibco, USA) with 10% fetal bovine serum and Iscove's Modified Dulbecco's Medium (Gibco, USA) supple`` mented with 20% fetal bovine serum, respectively. THP1 cells were cultured in RPMI 1640 medium supplemented with 0.05 mM 2-mercaptoethanol and 10% fetal bovine serum. RAW264.7 cell line and HEK-293T cell line were cultured in Dulbecco's Modified Eagle’s medium (Gibco, USA) supplemented with 10% fetal bovine serum. All cell lines were maintained at 37 °C in a humidified atmosphere at 5% CO2 and 95% air.

The THP-1 cells were differentiated into M0 macrophages by treatment with 100 nM PMA for 48h. Then, M0 macrophages were respectively incubated with 100 ng/mL lipopolysaccharide (LPS) and 20 ng/mL IFN-γ for further 48h to obtain M1-like macrophages, and with 50 ng/mL IL-4 and 20 ng/mL IL-13 for further 48h to obtain

M2-like macrophages. To obtain the TAMs, M0 macrophages were co-cultured with conditioned media (CM) from GC cell lines (NCI-N87 and KATO III) for another 48h.

Plasmid construction and stable cell line establishment

The pLVX-IRES-puro and pISH plasmids were gifts from Professor Gang Ma (Department of Gastroenterology, Tianjin Medical University Cancer Institute and Hospital, Tianjin, China). The human CCL2 and mouse ZC3H12A genes were cloned into the pLVX-IRES-puro plasmid. The following shRNA sequences for ZC3H12A (mouse) were as follows：shRNA #1 (CCACACAGATATTACCGTGTT) and shRNA #2 (CAAAGAAAGGGCTGGTGTATA), and cloned into the pISH plasmids. Lentiviruses were produced in HEK-293T cells for pLVX-CCL2 plasmid, pLVX-ZC3H12A(mouse) plasmid and pLVX-empty vector, as well as pISH-ZC3H12A-shRNA#1 plasmid, pISH-ZC3H12A-shRNA#2 plasmid and pISH-empty vector.

To obtain the stable transfection of cell lines, 1 × 105 cells were infected with 1 ml lentivirus supernatant with 20 μg/ml polybrene, and then selected from puromycin treatment. The pCDNA3.1-ZC3H12A-Myc (human ZC3H12A, NM_025079), pCDNA3.1-TRAF6-Flag (human TRAF6, NM_004620), pCDNA3.1-TRAF3-Flag (human TRAF3, NM_003300), pCDNA3.1-CD40-His (human CD40, NM_001250), pCDNA3.1-UB-K63 (human UB, all lysine mutated to arginine, except for K63), and pCDNA3.1-UB-K48 (human UB, all lysine mutated to arginine, except for K48) were manufactured and purchased from Synbio techoloies Co., Ltd, China. The pET28a(+)-CD40×HER2 bsAb plasmid was used to express the CD40×HER2 bsAb and the

sequence was describe in our previous study [2]. All plasmids were sequence-verified. Plasmids were transfected with Lipofectamine TM 2000 transfection reagent (Invitrogen, USA) and Opti-MEM (Invitrogen, USA).

RNA extraction and PCR analyses

Total RNA was extracted from cell lines by using Trizol reagent (Invitrogen, USA). Complementary DNA was synthesized by using HiScript III 1st Strand cDNA Synthesis Kit (Vazyme Biotech Co., Ltd, China) according to the manufacturer's instructions. For qPCR, ChamQ Universal SYBR qPCR Master Mix (Vazyme Biotech Co., Ltd, China) was used to test the gene expression. The primers used in this study were listed in the **Supplementary Table 2**.

Western blot analyses

Total protein was extracted by the RIPA buffer supplemented with protease and phosphatase inhibitor cocktail (Thermo Fisher Scientific, USA). 15 ug of protein was separated by the SDS-PAGE and transferred onto nitrocellulose membrane. The primary antibodies in this study are shown in **Supplementary Table 3**.

Hematoxylin-eosin (H&E) and immunohistochemistry (IHC) staining

HE staining was performed in the patient tissues and the mouse tumor tissues. The paraffin-embedded sections of tissues were deparaffinized and then stained using the H&E staining kit (Solarbio Science & Technology Co., Ltd, China) according to the manufacturer's instructions. The pathological changes were observed under a light microscope. IHC staining was performed to exam the CCL2, CD68, CD86 and CD206

Sun W, *et al. J Immunother Cancer* 2022; 10:e005063. doi: 10.1136/jitc-2022-005063

expression in patient tumor tissues, and CCL2, caspase3, F480, CD86 and CD206 expression in mouse tumor tissues. In brief, the paraffin-embedded sections of tissues were deparaffinized and heated for 30 minutes to retrieve the antigens. The sections were incubated with primary antibodies (**Supplementary Table 3**) overnight at 4℃. The sections were incubated with secondary antibody for 30 minutes and then were stained by a DAB staining Kit. The intensity was scored as follows: 0, negative; 1, weak; 2, moderate; and 3, strong. The frequency of positive cells was defined as follow: 0, less than 5%; 1, 5% to 25%; 2, 26% to 50%; 3, 51% to 75%; and 4, greater than 75%. When the staining was heterogeneous, we scored it as follows: each component was scored independently and summed for the results.

Immunofluorescence

Immunofluorescence staining was performed to exam the CD68, and CD206 expression in patient tumor tissues. In brief, the paraffin-embedded sections of tissues were deparaffinized and heated for 30 minutes to retrieve the antigens. The sections were permeabilized with 0.3% Triton X-100 for 20 min, and blocked with 3% BSA for 30 minutes. Subsequently, the sections were incubated with anti-CD68 and anti-CD206 overnight at 4°C, followed by Alexa Fluor 488-conjugated mouse secondary antibody and Alexa Fluor 555-conjugated rabbit secondary antibody (Life Technologies Corporation, USA) for 1 h at room temperature. Then, after the nucleus was labeled with DAPI (Abcam), we observed the results using a fluorescence microscope (Olympus, Japan) at magnification 200×.

Flow cytometry

The in vitro TAMs induced from THP1 and mouse tumor tissues were processed into single cell suspensions. After Fc block incubation for 20min, cells were incubated with antibodies to stain the surface markers. Then, cells were then fixed and permeabilized with Fixation/Permeabilization Buffer Set (eBioscience, USA). Next, cells were incubated with antibodies to stain intracellular surface markers. Finally, flow cytometry was performed using a FACSCanto^{TM} II flow cytometer (BDBiosciences, USA). All antibodies used in this study were purchased from eBioscience or Biolegend, and listed in the **Supplementary Table 3.**

CCK8 assay

CCK8 assay was applied to evaluate the drug sensitivity and half inhibitory concentration (IC_{50}) of GC to the trastuzumab treatment. GC cells (2000 cells per well) and TAMs were seeded into 96-well plates in a ratio 10:1. The mixed cells were incubated with the 50μg/mL trastuzumab and measured once a day. 10ul of CCK8 solution was added to every single well and the absorbance was measured at 450nm. For IC_{50}, GC cells (4000 cells per well) and TAMs were seeded into 96-well plates in a ratio 10:1. The mixed cells were treated with the trastuzumab at gradient concentrations of from 0 to 1000 μg/mL. After 72h incubation, the plates were added with CCK8 solution and measured in at 450 nm in a spectrophotometer.

5-ethynyl-20-deoxyuridine (EdU) assay

GC cells were co-cultured with TAMs in a non-contact co-culture transwell system

Sun W, *et al. J Immunother Cancer* 2022; 10:e005063. doi: 10.1136/jitc-2022-005063

(Corning, USA). Then, the 50μg/mL trastuzumab were added into the co-culture system. After 72h incubation, the GC cells were stained with 5-ethynyl-20-deoxyuridine (EdU, Ribobio, China) according to the manufacturer's instruction. In brief, cells were exposed to the Edu solution for additional 4 hours. After incubation with 1×Apollo reaction cocktail, the cells were stained with 1×Hoechst 33342 and then observed under a fluorescent microscope.

CD40 signaling reporter assay

The human M0 macrophages induced from THP1 by PMA and the mouse RAW264.7 cells (5×10^5 per well) were seeded in 6-well plates. The next day, the cells were incubated with CD40×HER2 bsAb at gradient concentrations from 0 to 100μg/mL. 48h after culture, the cells were harvested and stained with PE anti-CD86 by flow cytometry assay. The fluorescence intensity was measured using the a FACSCantoTM II flow cytometer (BDBiosciences, USA).

Co-immunoprecipitation assay

HEK-293T cells were transfected with empty vetor, pCDNA3.1-TRAF6-Flag, pCDNA3.1-TRAF3-Flag, or pCDNA3.1- ZC3H12A-Myc plasmid. After culture for 48h, total proteins were extracted using NP-40 lysis buffer (Solarbio Science & Technology Co., Ltd, China). Anti-DYKDDDDK-Tag Magnetic Beads (Abmart, China) and anti-Myc-Tag Agarose Beads (Abmart, China) were adopted for collecting the immunoprecipitated proteins. The immunoprecipitated proteins were measured by the western blot analysis

Sun W, *et al. J Immunother Cancer* 2022; 10:e005063. doi: 10.1136/jitc-2022-005063

Statistical analysis

The statistical software package SPSS 24.0 was used for all analyses. Categorical data and continuous variables in the clinicopathological characteristics were analyzed by the $\chi 2$ test and Student's test, respectively. The mean and standard deviation were used to evaluate data. Overall survival (OS), first progression (FP) and post progression survival (PPS) was determined using Kaplan–Meier method, and log-rank test was performed to determine significance. Significance was defined as two-side $P<0.05$.

Sun W, *et al. J Immunother Cancer* 2022; 10:e005063. doi: 10.1136/jitc-2022-005063

References:

[1] Lanczky A, Gyorffy B. Web-Based Survival Analysis Tool Tailored for Medical Research (KMplot): Development and Implementation [J]. J Med Internet Res, 2021, 23(7): e27633.

[2] Lu L, Liu N, Fan K, Zhang G, Li C, Yan Y, Liu T, Fu WH. A tetravalent single chain diabody (CD40/HER2) efficiently inhibits tumor proliferation through recruitment of T cells and anti-HER2 functions [J]. Mol Immunol, 2019, 109(149-56.
