# Supplementary material for: CD40×HER2 bispecific antibody overcomes the CCL2-induced trastuzumab resistance in HER2-positive gastric cancer
Source: J Immunother Cancer. 2022 Jul 15;10(7):e005063. doi: 10.1136/jitc-2022-005063 (PMC9295658; doi:10.1136/jitc-2022-005063)
Supplement: Supplementary data [file jitc-2022-005063supp010.pdf]

Supplementary Table 2. Real-time PCR primers used in this study

Gene	Primer type	Sequence (5' to 3')
CCL2 (human)	Forwrd	CAGCCAGATGCAATCAATGCC
CCL2 (human)	Reverse	TGGAATCCTGAACCCACTTCT
CD68 (human)	Forwrd	CTTCTCTCATTCCCCTATGGACA
CD68 (human)	Reverse	GAAGGACACATTGTACTCCACC
CD14 (human)	Forwrd	GACCTAAAGATAACCGGCACC
CD14 (human)	Reverse	GCAATGCTCAGTACCTTGAGG
CD36 (human)	Forwrd	CTTTGGCTTAATGAGACTGGGAC
CD36 (human)	Reverse	GCAACAAACATCACCACACCA
CD86 (human)	Forwrd	CCATCAGCTTGTCTGTTTCATTCC
CD86 (human)	Reverse	GCTGTAATCCAAGGAATGTGGTC
iNOS (human)	Forwrd	TTCAGTATCACAACCTCAGCAAG
iNOS (human)	Reverse	TGGACCTGCAAGTTAAAATCCC
TNF-α (human)	Forwrd	GAGGCCAAGCCCTGGTATG
TNF-α (human)	Reverse	CGGGCCGATTGATCTCAGC
ARG1 (human)	Forwrd	TGGACAGACTAGGAATTGGCA
ARG1 (human)	Reverse	CCAGTCCGTCAACATCAAAACT
CD204 (human)	Forwrd	GGTTTCAATTGTAAAGAGAGAGAAG
CD204 (human)	Reverse	CTGAGCAATTCTTCGTTTCCC
CD206 (human)	Forwrd	GGGTTGCTATCACTCTCTATGC
CD206 (human)	Reverse	TTTCTTGTCTGTTGCCGTAGTT
ZC3H12A (human)	Forwrd	TTCCTGCGTAAGAAGCCACTC
ZC3H12A (human)	Reverse	GAATCGGCACTTGATCCCATAG
ZC3H12B (human)	Forwrd	GCCAGCTCTGTCGATCACC
ZC3H12B (human)	Reverse	GCTTTAGCGCAAACTCCATCTT
ZC3H12C (human)	Forwrd	CAGCAAAGTGGAGTCAAGTACA
ZC3H12C (human)	Reverse	GCTGGACTTAACTGTGGGTCA
ZC3H12D (human)	Forwrd	AGTTCTCTGCGACCCATAGTG
ZC3H12D (human)	Reverse	AACAGCCAGCTTGATTCCCC
GAPDH (human)	Forwrd	CTCCTCCACCTTTGACGCTG
GAPDH (human)	Reverse	TCCTCTTGTGCTCTTGCTGG
ZC3H12A (mouse)	Forwrd	ACGAAGCCTGTCCAAGAATCC
ZC3H12A (mouse)	Reverse	TAGGGGCCTCTTTAGCCACA
ZC3H12B (mouse)	Forwrd	CCCAGAGCAGCATTTTACAGG
ZC3H12B (mouse)	Reverse	TCTGCATAACCCAACTTTAGAGC
ZC3H12C (mouse)	Forwrd	CTCTGAGGGAAGCACGAGTTC
ZC3H12C (mouse)	Reverse	GAGCGACATAGCTGTCTGTGA
ZC3H12D (mouse)	Forwrd	ACATCAAGGTTTTTGTCCCATCT
ZC3H12D (mouse)	Reverse	GGTCATCGTAGCAGACCACTC
GAPDH (mouse)	Forwrd	CTCCTCCACCTTTGACGCTG
GAPDH (mouse)	Reverse	TCCTCTTGTGCTCTTGCTGG
