# Supplementary material for: CD40×HER2 bispecific antibody overcomes the CCL2-induced trastuzumab resistance in HER2-positive gastric cancer
Source: J Immunother Cancer. 2022 Jul 15;10(7):e005063. doi: 10.1136/jitc-2022-005063 (PMC9295658; doi:10.1136/jitc-2022-005063)
Supplement: Supplementary data [file jitc-2022-005063supp011.pdf]

Supplementary Table 3. Main antibodies adopted in study

Name	Company	Cat. No.	Application
Anti-CCL2	R&D	MAB679	WB (Hu,1:1000),<br>IHC (Hu, Mo, 1:200)
Anti-ZC3H12A	Proteintech	25009-1-AP	WB (Hu,1:2000)
Anti-CD40	R&D	AF632	In vitro agonist activity
Anti-CD40	Bio X Cell	BE0016-2	In vivo agonist activity
Anti- iNOS	Santa Cruz	sc-7271	WB (Hu, 1:200)
Anti-Arg1	CST	#93668	WB (Hu, 1:1000)
Anti-p-p65 (Ser536)	CST	#3033	WB (Hu, 1:1000)
Anti-total-p65	CST	#8242	WB (Hu, 1:1000)
Anti-p-IKKβ (Ser176/180)	CST	#2694	WB (Hu, 1:1000)
Anti- total-IKKβ	Abcam	ab124957	WB (Hu, 1:1000)
Anti- p-IκBα (Ser32/36)	CST	#9246	WB (Hu, 1:1000)
Anti-total-IκBα	CST	#9242	WB (Hu, 1:1000)
Anti-Flag/ DYKDDDDK-tag	Abmart	M20008	WB (Hu, 1:5000)
Anti-Myc-tag	Abmart	M20002	WB (Hu, 1:5000)
Anti-His-tag	Abmart	M20001	WB (Hu, 1:5000)
Anti-Flag-tag (HRP Conjugated)	Abmart	M20026	WB (Hu, 1:5000)
Anti-Myc-tag (HRP Conjugated)	Abmart	M20019	WB (Hu, 1:1000)
Anti-His-tag (HRP Conjugated)	Abmart	M20020	WB (Hu, 1:5000)
Anti-HA (HRP Conjugated)	Abmart	M20021	WB (Hu, 1:5000)
Anti-β-actin	Proteintech	66009-1-Ig	WB (Hu, 1:1000)
Anti-F4/80	novus	NB600-404	IHC (Mo, 1:500)
Anti-CD68	Abacam	ab955	IHC (Hu, 1:1000)<br>IF (Hu,1:500)
Anti-CD86	novus	NBP2-25208	IHC (Mo,1:500)
Anti-CD86	Santa Cruz	sc-28347	IHC (Hu, 1:200)
Anti-CD206	Abacam	ab64693	IHC (Hu, Mo, 1:500)<br>IF (Hu, 1:100)
Caspase 3	CST	#9662	IHC (Mo, 1:1000)
TruStain Fc X	Biolegend	422302	Flow cytometry (Hu)
FITC anti-CD68	eBioscience	11-0689-42	Flow cytometry (Hu)
PE anti-CD86	eBioscience	12-0862-81	Flow cytometry (Hu)
APC anti-CD206	Biolegend	321110	Flow cytometry (Hu)
Anti-CD16/CD32	eBioscience	14-0161-82	Flow cytometry (Mo)

PE/Cy7 anti-CD45	Biolegend	103114	Flow cytometry (Mo)
APC anti-CD11b	eBioscience	17-0112-81	Flow cytometry (Mo)
FITC anti-F4/80	eBioscience	11-4801-82	Flow cytometry (Mo)
PE anti-CD206	eBioscience	12-2061-80	Flow cytometry (Mo)
