# Supplementary figures and images for: CD40×HER2 bispecific antibody overcomes the CCL2-induced trastuzumab resistance in HER2-positive gastric cancer
Source: J Immunother Cancer. 2022 Jul 15;10(7):e005063. doi: 10.1136/jitc-2022-005063 (PMC9295658; doi:10.1136/jitc-2022-005063)

Supplementary Figure 1

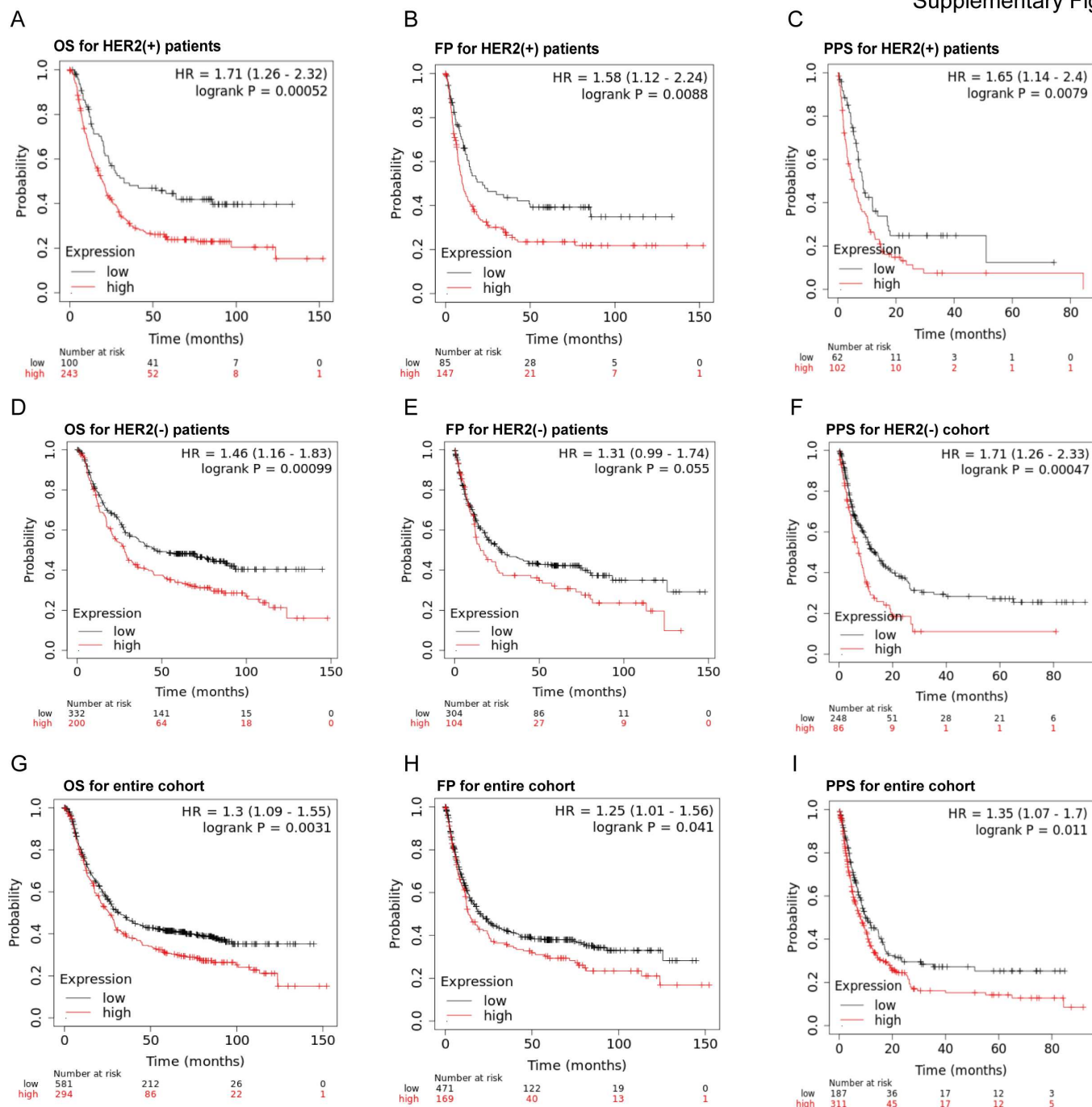

Supplement: Supplementary data [file jitc-2022-005063supp002.pdf]

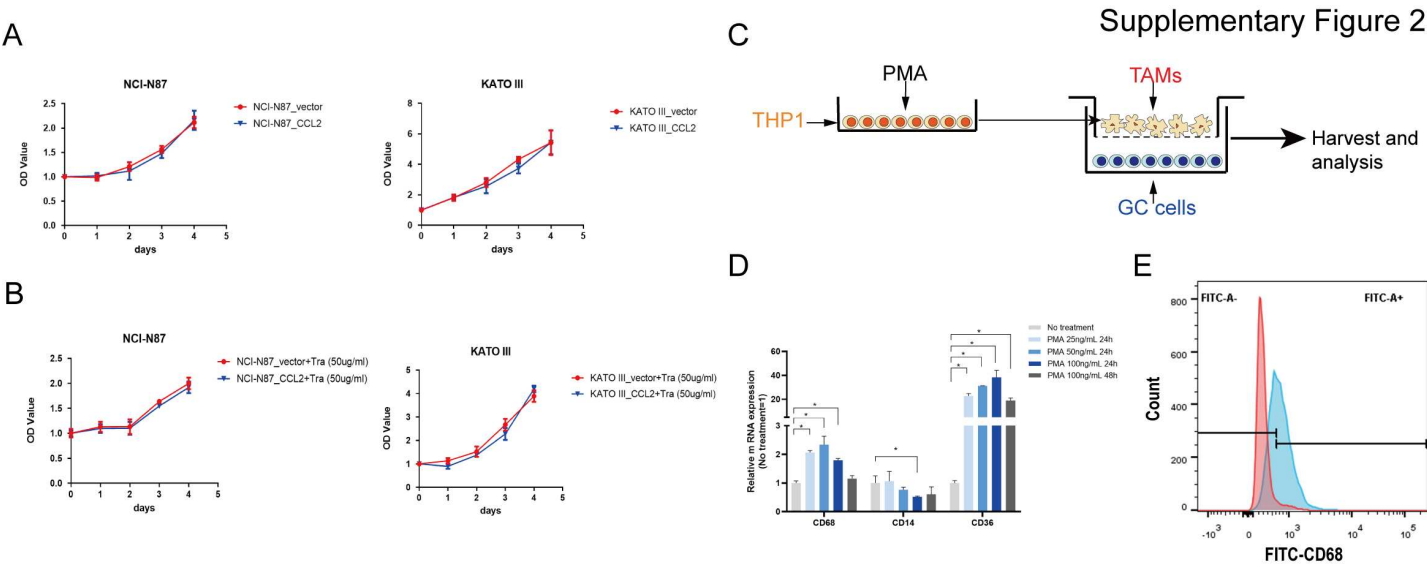

Supplement: Supplementary data [file jitc-2022-005063supp003.pdf]

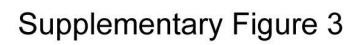

Supplement: Supplementary data [file jitc-2022-005063supp004.pdf]

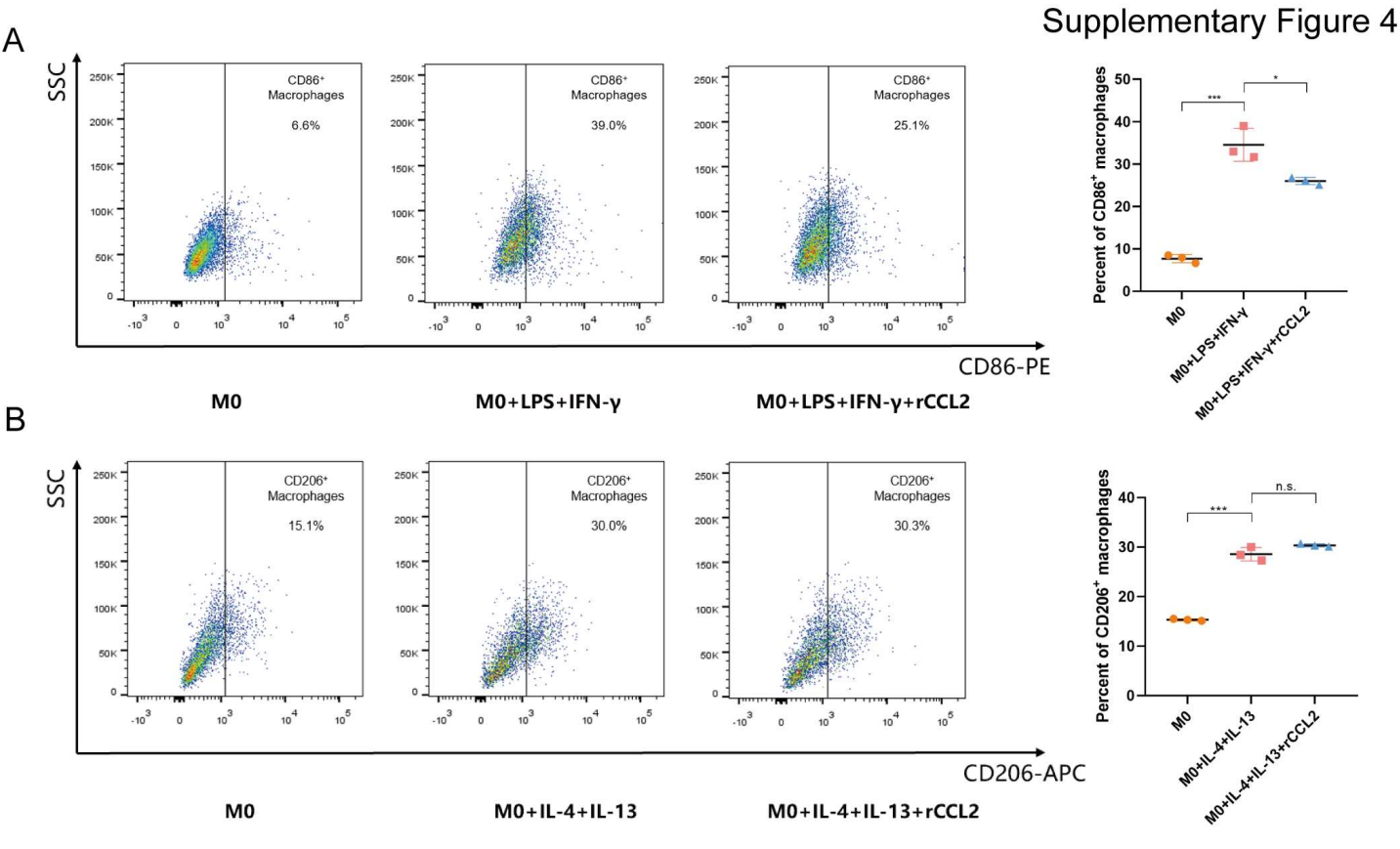

Supplement: Supplementary data [file jitc-2022-005063supp005.pdf]

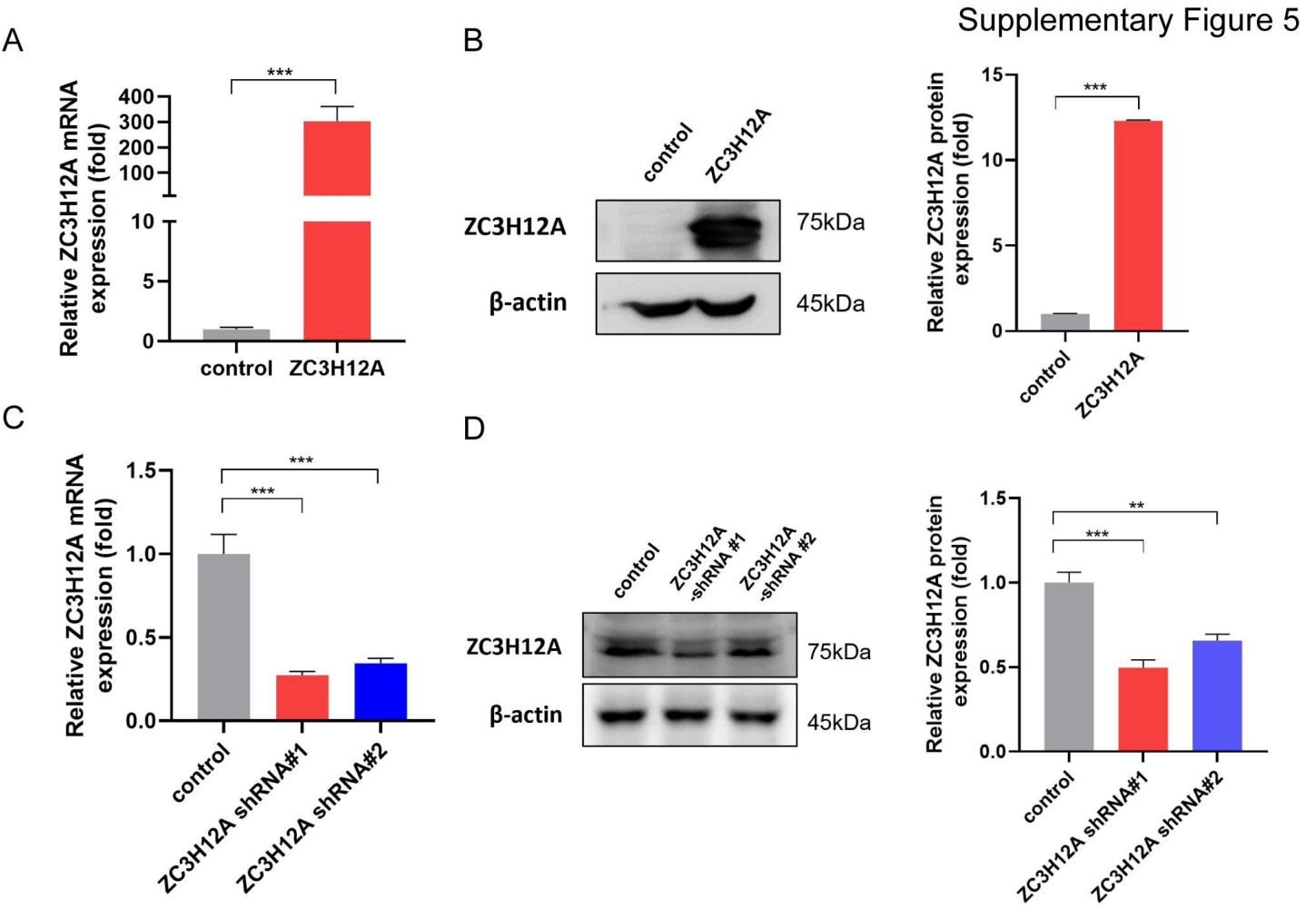

Supplement: Supplementary data [file jitc-2022-005063supp006.pdf]

Supplementary Figure 6

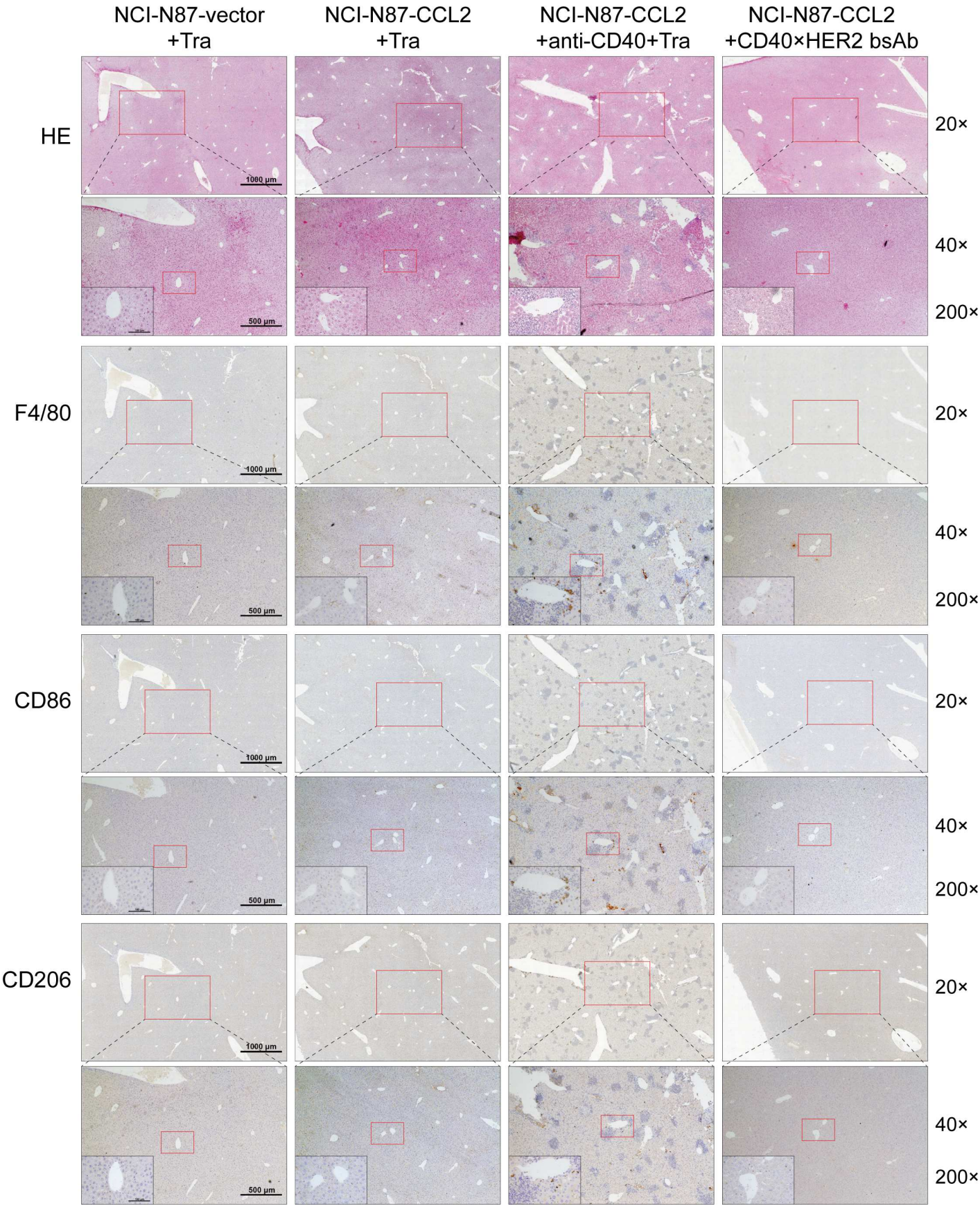

Supplement: Supplementary data [file jitc-2022-005063supp007.pdf]

Supplementary Figure 7

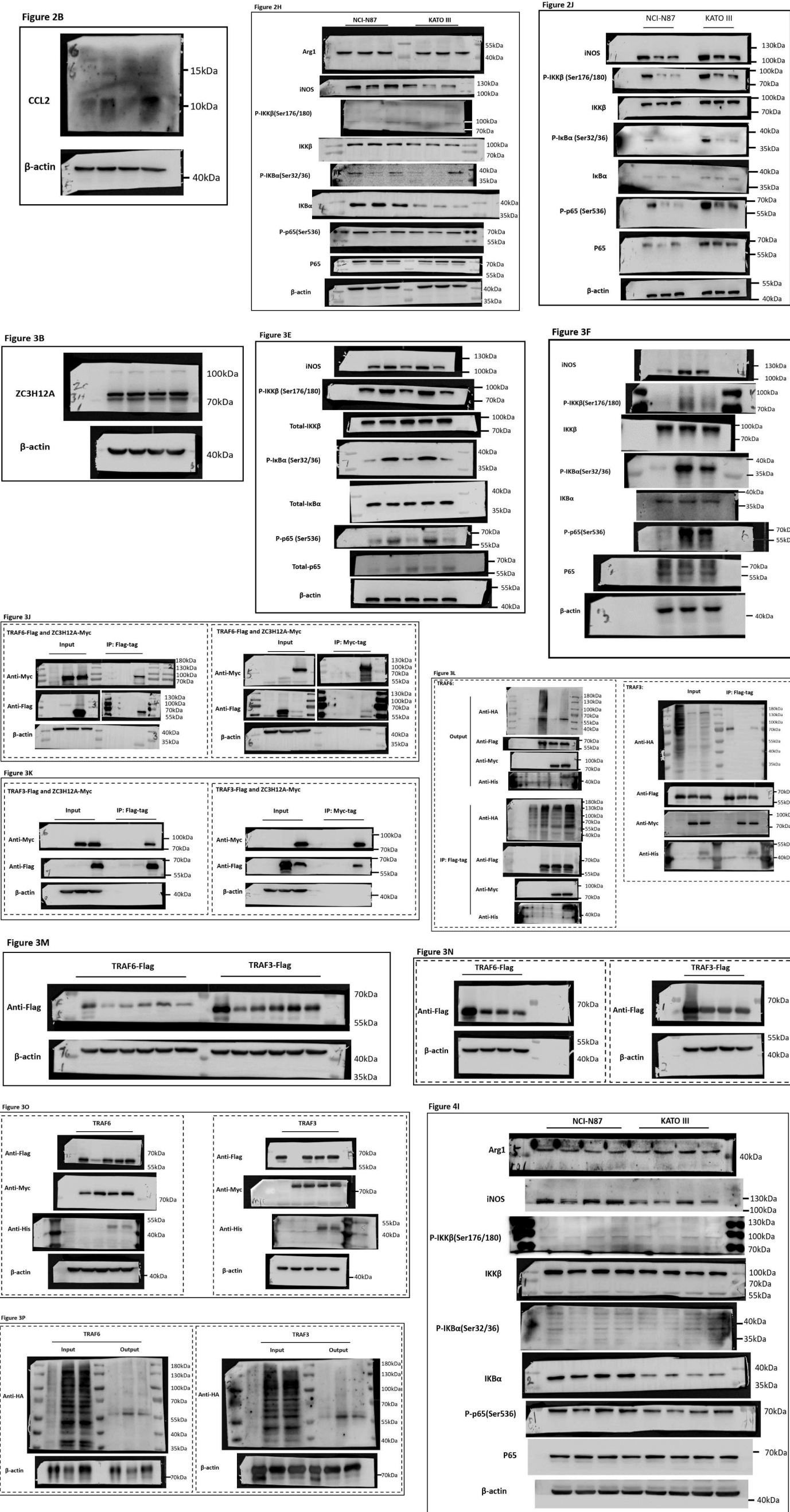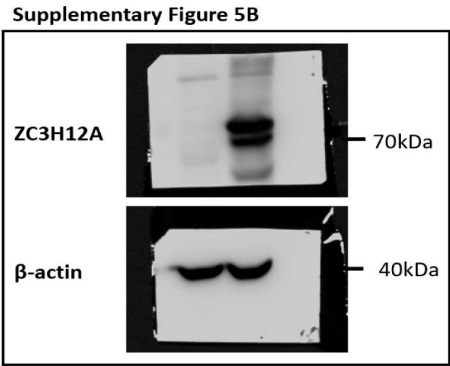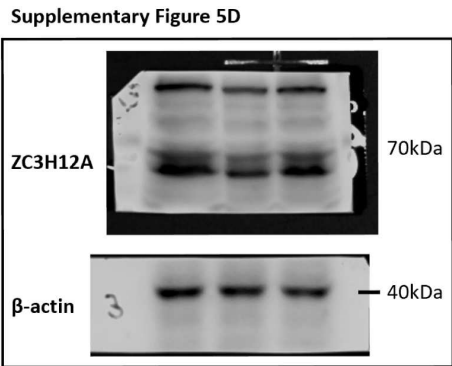

Supplement: Supplementary data [file jitc-2022-005063supp008.pdf]
